# Crosstalk Between CTSB^+^ Glioblastoma Cells and S100A10^+^ Macrophages: A Self‐Reinforcing Circuit Promotes Immune Evasion and Limits Response to Immunotherapy

**DOI:** 10.1002/advs.76905

**Published:** 2026-08-29

**Authors:** Hao Zhang, Nan Zhang, Xisong Liang, Jie Wen, Ziyu Dai, Wantao Wu, Shuyu Li, Songshan Feng, Zaoqu Liu, Zhiwei Xia, Peng Luo, Quan Cheng

**Affiliations:** ^1^ Department of Neurosurgery Xiangya Hospital Central South University Changsha China; ^2^ Department of Neurosurgery The Second Affiliated Hospital Chongqing Medical University Chongqing China; ^3^ National Clinical Research Center for Geriatric Diseases (Xiangya Hospital) Central South University Changsha China; ^4^ Western Institute of Digital‐Intelligent Medicine Chongqing China; ^5^ Department of Obstetrics and Gynecology National Clinical Research Center for Obstetrics and Gynecology Tongji Hospital Tongji Medical College Huazhong University of Science and Technology Wuhan China; ^6^ Key Laboratory of Cancer Invasion and Metastasis (Ministry of Education) Hubei Key Laboratory of Tumor Invasion and Metastasis Tongji Hospital Tongji Medical College Huazhong University of Science and Technology Wuhan China; ^7^ College of Life Science and Technology Huazhong University of Science and Technology Wuhan China; ^8^ Department of Thyroid and Breast Surgery The Second Affiliated Hospital Chongqing Medical University Chongqing China; ^9^ Department of Thyroid and Breast Surgery Tongji Hospital Tongji Medical College of Huazhong University of Science and Technology Wuhan China; ^10^ Institute of Basic Medical Sciences Chinese Academy of Medical Sciences and Peking Union Medical College Beijing China; ^11^ Department of Neurology Hunan Aerospace Hospital Hunan Normal University Changsha China; ^12^ Department of Oncology Zhujiang Hospital Southern Medical University Guangzhou China; ^13^ Department of Neurosurgery Xiangya Hospital Central South University (National Regional Center For Neurological Diseases) Nanchang China; ^14^ Hypothalamic Pituitary Research Centre Xiangya Hospital Central South University Changsha China

**Keywords:** CTSB, glioblastoma, immunotherapy, S100A10, tumor‐associated macrophage

## Abstract

**Background**: Glioblastoma (GBM) establishes a highly immunosuppressive microenvironment through intricate molecular dialogues with tumor‐associated macrophages (TAMs), contributing to immunotherapy resistance.

**Methods**: We integrated advanced biological network analyses with multimodal AI to identify CTSB as a key biomarker. Functional validation included GBM‐macrophage co‐culture systems, chromatin immunoprecipitation (ChIP), dual‐luciferase reporter assays, molecular docking, and co‐immunoprecipitation (Co‐IP). In vivo experiments employed orthotopic (GL261 and CT‐2A) and subcutaneous GBM models with lentivirus‐mediated CTSB knockdown and anti‐PD‐1 therapy. Tumor microenvironment dynamics were assessed via in‐house generated single‐cell RNA sequencing and multiplex immunofluorescence.

**Results**: Cathepsin B (CTSB) was identified as a critical driver of aggressive GBM progression and poor outcomes. Mechanistically, macrophage‐derived IL‐6 activates STAT3 in tumor cells, upregulating CTSB expression. Structural and Co‐IP analyses revealed CTSB, secreted by tumor cells, binds the C‐terminus of macrophage S100A10, reinforcing IL‐6 secretion, forming a feedforward loop between CTSB^+^ GBM cells and S100A10^+^ macrophages. This loop enhances tumor growth, invasion, and immune evasion via PD‐L1 upregulation. In vivo, CTSB blockade reduced TAM activation, increased CD8^+^ T cell infiltration, and synergized with anti‐PD‐1 therapy.

**Conclusions**: This study unveils a targetable GBM‐macrophage signaling axis, proposing CTSB inhibition as a strategy to enhance immunotherapy efficacy in GBM patients.

## Graphical abstract

In glioblastoma, M2‐polarized macrophages secrete IL‐6, which activates STAT3 signaling in tumor cells to upregulate CTSB. Tumor‐derived CTSB binds the C‐terminus of macrophage S100A10, reinforcing M2 polarization and further IL‐6 secretion, thereby establishing a feedforward IL‐6/STAT3/CTSB/S100A10 loop. This cascade drives tumor growth, invasion, and immune suppression, ultimately conferring resistance to anti‐PD‐1 immunotherapy. Blockade of CTSB disrupts this positive feedback loop, reduces M2 macrophage activation, enhances CD8+ T cell infiltration, and synergizes with anti‐PD‐1 therapy, representing a promising therapeutic strategy for glioblastoma.

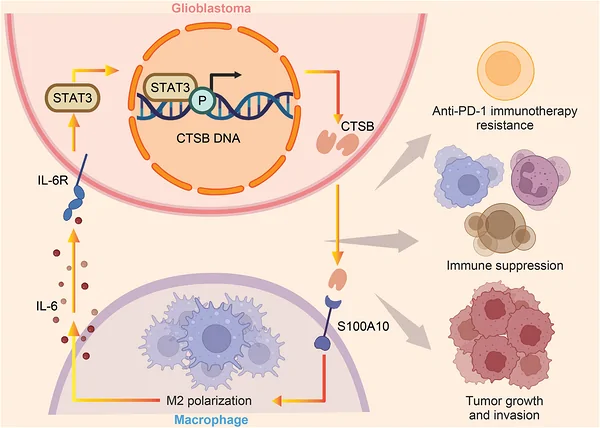

**Abbreviations**
AyersExpIS: Expanded Immune Signature
CCK‐8: Cell Counting Kit‐8
CGGA: Chinese Glioma Genome Atlas
ChIP: Chromatin Immunoprecipitation
Co‐IP: Co‐Immunoprecipitation
CTSB: Cathepsin B
CYT: Immune Cytolytic Activity
DavoliIS: Davoli Immune Signature
DEG: Differentially Expressed Gene
EdU: 5‐Ethynyl‐2’‐Deoxyuridine
ELISA: Enzyme‐Linked Immunosorbent Assay
ESTIMATE: Estimation of Stromal and Immune Cells in Malignant Tumor Tissues Using Expression
GBM: Glioblastoma
GEO: Gene Expression Omnibus
GEP: T Cell‐Inflamed Signature
GO: Gene Ontology
GRAMM: Global Range Molecular Matching
GSEA: Gene Set Enrichment Analysis
HE: Hematoxylin and Eosin
ICB: Immune Checkpoint Blockade
ICBnetIS: Network‐Based ICB Immunotherapeutic Signature
IFNγIS: Interferon‐Gamma Immune Signature
IHC: Immunohistochemistry
IL‐6: Interleukin‐6
ImmPort: Immunology Database and Analysis Portal
KEGG: Kyoto Encyclopedia of Genes and Genomes
LassoCox: Least Absolute Shrinkage and Selection Operator Regularized Cox Regression
MEGENA: Multiscale Embedded Gene Co‐expression Network Analysis
mIF: Multiplex Immunofluorescence

## 1 Introduction

Glioblastoma multiforme (GBM) remains the most aggressive and lethal primary malignant brain tumor in adults, with a dismal median overall survival of less than 15 months despite maximal surgical resection, radiotherapy, and temozolomide‐based chemotherapy [1]. The pervasive treatment resistance and high recurrence rate of GBM are largely attributed to its highly heterogeneous nature, invasive growth pattern, and, most critically, an extremely immunosuppressive tumor microenvironment [2]. Immunotherapy, with its efficacy affected by tumor microenvironment, has emerged as a promising therapeutic option for solid cancer types, such as melanoma and lung cancer [3]. However, immunotherapy only benefits a limited fraction of GBM patients [1]. Its resistance to immunotherapy [4] highlights the urgent need to decode mechanisms by which GBM manipulates its microenvironment.

Tumor‐associated macrophages (TAMs) are the most abundant immune stromal cells in GBM, constituting up to 30%–50% of the tumor mass, and their high infiltration correlates tightly with tumor grade, invasive progression, and poor patient prognosis [5]. Unlike anti‐tumor M1 macrophages, TAMs in GBM predominantly exhibit an M2‐like immunosuppressive phenotype, which fosters tumor angiogenesis, extracellular matrix remodeling, and suppression of cytotoxic T‐cell infiltration and function [6]. Recent advances in tumor microenvironment biology have revealed that feedforward signaling loops between cancer cells and TAMs create self‐reinforcing ecosystems [7]. While GBM‐derived factors like exosomes [8] and cytokines [9] promote tumor‐associated macrophage activation, the molecular mediators sustaining this pathogenic crosstalk remain poorly defined.

Against this background, identifying key molecular mediators that govern the GBM–TAM crosstalk and dissecting their underlying regulatory mechanisms are of paramount importance to reverse immunosuppression and restore immunotherapy sensitivity. With the rapid advancement of high‐throughput omics technologies and artificial intelligence‐driven bioinformatic analyses, integrative multi‐dimensional profiling of large‐scale clinical cohorts has become a powerful strategy to uncover disease‐driving hub genes and actionable therapeutic targets [10, 11, 12]. By combining biological network inference, machine learning‐based feature selection, and survival modeling, researchers can systematically prioritize functionally critical genes from complex transcriptomic landscapes that are closely associated with TAM activity, immune suppression, and patient prognosis.

Here, this study employed multi‐dimensional analyses across large‐scale IDH wild‐type (IDHWT) GBM samples with functional validation to identify Cathepsin B (CTSB) as a keystone molecule in GBM‐macrophage symbiosis and a critical hazardous marker in GBM prognosis. It was demonstrated that macrophage‐derived Interleukin‐6 (IL‐6) activates STAT3 to transcriptionally upregulate CTSB in tumor cells, which in turn binds to the C‐terminus of S100A10 on macrophages to reinforce their IL‐6 secretion, creating a pathogenic feedback loop. Clinical specimens showed spatial co‐localization of CTSB^+^ cells with S100A10^+^ TAMs, while preclinical models establish CTSB targeting as a strategy to remodel the immunosuppressive niche and potentiate checkpoint blockade. These findings reveal an actionable axis at the tumor‐macrophage interface that perpetuates GBM's malignant ecology, with immediate translational potential.

## 2 Materials and Methods

### 2.1 Public Data Collection and Processing

IDHWT GBM samples were collected from The Cancer Genome Atlas (TCGA), Chinese Glioma Genome Atlas (CGGA), and Gene Expression Omnibus (GEO) databases. TCGAseq‐IDHWT cohort, TCGAarray‐IDHWT cohort, CGGA‐IDHWT cohort, GSE16011‐IDHWT, and GSE108474‐IDHWT were integrated into the meta‐IDHWT cohort. The data normalization and processing procedure followed the previous study [13].

### 2.2 Identification of the TAM‐related Genes by Biological Network Analyses

The STRING [14] and HINT [15] databases were combined to generate the protein‐protein interaction (PPI) network (21177 genes). The Random Walk with Restart (RWR) algorithm [16], a widely used network‐based ranking method, was applied to estimate the association strength between each gene in the PPI network and TAMs. The analysis was initiated using the 19 predefined TAM‐specific genes [17] as starting seed nodes, allowing stepwise propagation of interaction weights across the network. The RWR formula is defined as follows:

$$P_{t+1}=\left(1-\;r\right)MP_{t}+rP_{0}$$

where M stands for the PPI network's sparse adjacency matrix, *P*_0_ is the preliminary probability distribution in which the TAM‐specific genes are assigned equal probabilities other than zero as starting seed nodes, *P_t_* represents the probability vector for the network nodes at step *t*, *r* is the restarting probability, which is set to 0.8. The walker repeatedly jumps between the current nodes and a set of neighboring nodes that are chosen randomly or back to the starting nodes. After repeated iterations, a stationary probability distribution is achieved, and the gap between the vectors *P*_*t* + 1_ and *P_t_* becomes negligible. The iterations were performed in this research until the difference between *P*_*t* + 1_ and *P_t_* was less than 10^−30^ as in a previous study [18]. Genes in the PPI network were sorted by their final probability scores, from highest to lowest, with higher‐ranked genes exhibiting stronger associations with TAMs. TAM‐associated PPIRWR‐derived gene ranking vectors were subjected to gene set enrichment analysis (GSEA) [19] via Gene Ontology (GO) terms [20]. A TAM score was calculated using the single‐cell gene set enrichment analysis (ssGSEA) method based on the 19 TAM‐specific genes. Survival curves for overall survival (OS) of the two TAM score‐stratified groups were generated using the R package survminer. Weighted gene co‐expression network analysis (WGCNA) [21] was performed to identify the TAM score‐related gene module from the top 10% of genes in the TAM‐associated PPIRWR‐derived gene ranking. Multiscale Embedded Gene Co‐expression Network Analysis (MEGENA) [22] was subsequently used to refine the WGCNA‐derived turquoise module, resolving a more condensed subnetwork associated with TAM and identifying critical hub genes.

### 2.3 Identification of the TAM‐Related Genes by Machine Learning

The consensus clustering method, partitioning around medoids (PAM) [23], was applied to determine TAM‐related patterns based on the 19 TAM‐specific genes. To evaluate immunological activity, computational analysis of pathways linked to immunity was performed using the Immunology Database and Analysis Portal (ImmPort) [24]. Infiltrating immune cell fractions were quantified through the Tumor Immune Estimation Resource (TIMER) algorithm [25]. The Pathway RespOnsive GENes (PROGENy) algorithm [26] was employed to estimate the activity of pathways linked to tumorigenesis. The R package limma was used to determine the differentially expressed genes (DEGs) between two TAM‐patterns [27]. Seven machine learning (ML) algorithms for classification, including Boruta [28], GlmBoost [29], least absolute shrinkage and selection operator regularized logistic regression (LassoLR) [30], prediction analysis for microarrays (Pamr) [31], Random Forest (RF) [32], support vector machine (SVM) [33], and XGBoost [34] were used to optimize the DEGs. Univariate Cox proportional hazard regression analysis was performed on classification‐based ML‐derived genes, and hazard ratios and 95% confidence intervals were estimated using the R package survival. Six ML algorithms for survival, including CoxBoost [35], least absolute shrinkage and selection operator regularized Cox regression (LassoCox) [36], StepwiseCoxForward, StepwiseCoxBoth, StepwiseCoxBackward, and Random Survival Forest (RSF) [37], were further used to optimize the prognostic genes.

### 2.4 Functional Annotation and Immune Features of CTSB

To explore the functional role of CTSB, enrichment analysis of CTSB‐associated genes was performed for GO terms and KEGG (Kyoto Encyclopedia of Genes and Genomes) pathways [38]. The cancer immune cycle [39] and the cancer immunogram traits [40, 41] were quantified. The abundance of stromal and immune cells was assessed using the Estimation of Stromal and Immune cells in Malignant Tumor tissues using Expression (ESTIMATE) algorithm [42]. Immune infiltration was assessed using three computational methods: 28 immune cell types were quantified with ssGSEA, following Pornpimol's framework [43], 10 immune cell types were measured by the MCP‐counter tool [44], and 6 immune cell types were analyzed with the TIMER algorithm [25]. The following ten immunotherapeutic signatures which effectively predict immune checkpoint blockade (ICB) therapy response were collected: immune cytolytic activity (CYT) [45], interferon‐gamma immune signature (IFNγIS) [46], expanded immune signature (AyersExpIS) [46], T cell‐inflamed signature (GEP) [46], Roh immune score (RohIS) [47], Davoli immune signature (DavoliIS) [48], network‐based ICB immunotherapeutic signature (ICBnetIS) [49], repressed immune resistance (RIR) [50], tertiary lymphoid structure (TLS) [51], and ImmuneScore generated by ESTIMATE [42].

### 2.5 Cell Culture

U251, T98G, GL261, THP‐1, and RAW 264.7 cell lines were purchased from the Cellverse Co., Ltd, Shanghai, China. CT‐2A cell line was donated by Beijing Tiantan Hospital, Capital Medical University. U251, T98G, GL261, and CT‐2A cells were cultured in DMEM medium (D5796, Sigma, USA) supplemented with 10% fetal bovine serum (FBS, Gibco) and streptomycin/penicillin (AWH0529, Abiowell, China). Luc‐GL261 cells and luc‐CT‐2A cells were obtained by infecting cells with a firefly luciferase reporter for bioluminescence tracking. THP‐1 and RAW 264.7 cells were cultured in RPMI‐1640 medium (R8758, Sigma, USA) with 10% FBS, respectively. THP‐1 cells were polarized into M0 macrophages after adding Phorbol 12‐myristate 13‐acetate (PMA) for 6 h and further polarized into TAMs after adding IL‐4 and IL‐13 for 72 h. RAW 264.7 cells were polarized into TAMs after adding IL‐4 for 12 h.

### 2.6 Co‐Culture System

U251 and T98G cells were resuspended at a density of 1 × 10^5^/mL and were added to the lower chamber. THP‐1‐polarized TAMs were resuspended at a density of 1 × 10^5^/mL, and a 100 µL suspension of TAMs was added to the upper chamber. After coculturing with TAMs for 48 h, U251 and T98G cells were collected for western blot assay, Cell counting kit‐8 (CCK‐8) assay, 5‐ethynyl‐2′‐deoxyuridine (EdU) assay, and flow cytometry‐based apoptosis detection. THP‐1‐polarized TAMs were resuspended at a density of 1 × 10^5^/mL and were added to the lower chamber. U251 and T98G cells were resuspended at a density of 1 × 10^5^/mL, and a 100 µL suspension of U251 and T98G cells was added to the upper chamber for the Transwell assay. Likewise, U251 and T98G cells were resuspended at a density of 1 × 10^5^/mL and were added to the lower chamber. THP‐1‐polarized TAMs were resuspended at a density of 1 × 10^5^/mL, and a 100 µL suspension of TAMs was added to the upper chamber for the Transwell assay. THP‐1‐polarized TAMs were resuspended at a density of 1 × 10^5^/mL and were added to the lower chamber. U251 and T98G cells were resuspended at a density of 1 × 10^5^/mL, and a 100 µL suspension of U251 and T98G cells was added to the upper chamber. After coculturing with U251 and T98G cells for 48 h, TAMs were collected for flow cytometry‐based polarization detection.

### 2.7 Cell Transfection

CTSB siRNA and control siRNA were purchased from the Sangon Biotech Co., Ltd. (Shanghai, China). U251 and T98G cells were added to 12‐well plates. 95 µL serum‐free DMEM was added to each of the two centrifuge tubes. 5 µL siRNA and 5 µL Lipofectamine 2000 Transfection Reagent (ThermoFisher, USA) were added to two centrifuge tubes for 5 min, respectively. Two centrifuge tubes were mixed for 20 min and added to 12‐well plates. The RNA oligo synthesis sequences of siRNA targeting human CTSB and control siRNA are listed in Table S1. S100A10 siRNA and control siRNA were purchased from the Sangon Biotech Co., Ltd. (Shanghai, China). THP‐1‐polarized M0 macrophages were added to 12‐well plates. The transfection protocol was the same as mentioned above. The RNA oligo synthesis sequences of siRNA targeting human S100A10 and control siRNA are listed in Table S2.

### 2.8 Plasmid Construction

The full‐length human STAT3 cDNA was subcloned into a pcDNA3.4 vector (C‐terminus with 3xMyc tag) to construct a STAT3‐overexpression plasmid (HonorGene, Changsha, China). STAT3‐overexpression plasmid was transfected into U251 and T98G cells with Lipofectamine 2000 Transfection Reagent (ThermoFisher, USA) according to the manufacturer's protocol.

The full‐length human S100A10 cDNA and its truncation variant (N79) were constructed and inserted into the pcDNA3.1‐Flag‐C vector (HonorGene, Changsha, China). The expression vector (pcDNA3.1‐His‐C) of CTSB was also constructed (HonorGene, Changsha, China). DNA transfection into THP‐1 polarized M0 macrophages was carried out using Lipofectamine 2000 according to the manufacturer's protocol.

### 2.9 Lentivirus Infection

The lentivirus (shRNA‐NC and shRNA‐Ctsb) was purchased from Genechem (Shanghai, China). The GL261 cells were infected with the lentivirus using 4 µg/mL polybrene, and stable cell lines were established by treating the infected cells with 5 µg/mL puromycin (ST551, Beyotime, China) for two weeks. The target sequences against murine Ctsb and control shRNA are listed in Table S3.

### 2.10 Western Blot Assay

To extract the total protein, tissues or cells were gathered and lysed using RIPA buffer, which contains protease inhibitors. Proteins in equal quantities were separated by electrophoresis using SDS‐PAGE gel and then transferred to PVDF membranes. After blocking the membranes for 2 h with 5% non‐fat dried milk in Tris‐buffered saline (TBS), the membranes were incubated with the primary and secondary antibodies for the entire night at 4°C.

### 2.11 Real‐Time Quantitative PCR

Total RNA was extracted with TRIzol reagent (15596026, ThermoFisher, USA), followed by cDNA synthesis employing the HiFiScript cDNA Synthesis Kit (CW2569, CWBIO, China). Real‐time quantitative PCR (RT‐qPCR) was performed using the UltraSYBR Mixture kit (CW2601, CWBIO, China), with primer sequences provided in Supplementary Table S4.

### 2.12 CCK‐8 Assay

U251 and T98G cells were resuspended at a density of 1 × 10^4^/mL and were added to 96‐well plates. CCK‐8 (NU679, DOJINDO, Japan) solution was added to 96‐well plates and cultured for 4 h. Optical density (OD) at 450 nm was measured using a microplate reader.

### 2.13 EdU Assay

U251 and T98G cells were resuspended and added to 6‐well plates. The EdU kit (C10310, Ribobio, China) was used to conduct the EdU assay. Following a 2‐h incubation period with 50 µM EdU solution, the cells were fixed for 30 min with 4% paraformaldehyde, decolored for 5 min with 2 mg/mL glycine, and treated for 10 min with 0.5% Triton X‐100. Following staining with Apollo and DAPI solutions, EdU‐positive cells were viewed and counted using a fluorescent microscope. The recombinant IL‐6 protein (HY‐P7044, MCE, USA) and IL‐6 monoclonal antibody (21865‐1‐AP, Proteintech, China) were used for cell intervention.

### 2.14 Transwell Assay

U251 and T98G cells were resuspended with serum‐free medium at a density of 1 × 10^5^/mL, and a 100 µL suspension of cells was seeded in the upper chamber of a Transwell chamber (3422, Corning, China). 500 µL DMEM with 10% FBS was added to the lower chamber. The migrated cells were counted after fixation in 4% paraformaldehyde and staining with 0.1% crystal violet. In the co‐culture system, the migrated co‐cultured U251 cells, T98G cells, and TAMs in the upper chamber were counted after fixation in 4% paraformaldehyde and staining with 0.1% crystal violet.

### 2.15 ELISA Assay

The protein expression of CTSB in the supernatants of U251 and T98G cells was detected using the ELISA (Enzyme‐Linked Immunosorbent Assay) kit (CSB‐E13450h, CUSABIO, China). The protein expression of IL‐6 in the supernatants of THP‐1 polarized TAMs was detected using the ELISA kit (KE00139, Proteintech, China).

### 2.16 Immunofluorescence Staining Assay

For THP‐1 polarized TAMs, CTSB (Rabbit, 12216‐1‐AP, Proteintech, China) and S100A10 (Rabbit, 11250‐1‐AP, Proteintech, China) were sequentially applied as primary antibodies, followed by horseradish peroxidase‐conjugated secondary antibody incubation (AWS0001, AWS0002, Abiowell, China) and tyramide signal amplification (TSA) using the staining kit (TSA‐520, TSA‐620, AWI0693a, Abiowell).

For U251 and T98G cells, IL‐6 (Rabbit, AWA46788, Abiowell, China) and IL‐6R (Rabbit, 23457‐1‐AP, Proteintech, China) were sequentially applied as primary antibodies, followed by horseradish peroxidase‐conjugated secondary antibody incubation (AWS0001, AWS0002, Abiowell, China) and tyramide signal amplification (TSA) using the staining kit (TSA‐520, TSA‐620, AWI0693a, Abiowell).

### 2.17 Flow Cytometry‐Based Apoptosis Detection

U251 and T98G cells were resuspended in 500 µL binding buffer, stained with 5 µL Annexin V‐APC and 5 µL Propidium Iodide (KGA1030‐1007, KeyGEN BioTECH, China) for 10 min in the dark. The apoptotic cells were assessed using flow cytometry (A00‐1‐1102, Beckman, USA).

### 2.18 Flow Cytometry‐Based Polarization Detection

THP‐1‐polarized M0 macrophages were resuspended in 500 µL intracellular fixation buffer for 30 min. After centrifugation, THP‐1‐polarized M0 macrophages were resuspended in 1 mL permeabilization buffer. After centrifugation again, THP‐1‐polarized M0 macrophages were resuspended in 100 µL permeabilization buffer and incubated with anti‐CD68 (12‐0689‐42, Invitrogen, USA) and anti‐CD163 (17‐1639‐42, Invitrogen, USA) antibodies in the dark for 30 min. The polarized cells were assessed using flow cytometry (A00‐1‐1102, Beckman, USA).

### 2.19 Chromatin Immunoprecipitation Assay

The binding between STAT3 and three different promoters of CTSB was validated by chromatin immunoprecipitation (ChIP) assay using the ChIP kit (ab500, Abcam, UK). Briefly, chromatin was extracted from U251, T98G, and LN229 cells, gently cross‐linked (1.1% formaldehyde, 10 min), sheared, and incubated with control IgG (anti‐IgG, Abcam ab171870) and STAT3 (10253‐2‐AP, Proteintech, China) antibodies overnight. Upon extensive washing, chromatin was eluted in DNA purifying slurry and treated with Proteinase K. Immunoprecipitated DNA was amplified by PCR.

### 2.20 Dual‐Luciferase Reporter Assay

Based on the binding sequence from the ChIP assay, the binding between STAT3 and the promoter of CTSB (CTSBp) was further validated by dual‐luciferase reporter assay. The pGL3‐CTSBp WT reporter or pGL3‐CTSBp MUT reporter was transfected into U251 cells with Lipofectamine 2000. The Renilla luciferase reporter (pRL‐TK) vector was used as a control. U251 cells were cultured in 24‐well plates and divided into five groups: pcDNA3.4+pGL3‐Basic (pcDNA3.4, pcDNA3.4‐STAT3‐3×Myc, pRL‐TK); pcDNA3.4+CTSB‐WT (pcDNA3.4, pGL3‐Basic‐CTSBp, pRL‐TK); STAT3+CTSBp‐WT (pcDNA3.4‐STAT3‐3×Myc, pGL3‐Basic‐CTSBp, pRL‐TK); pcDNA3.4+ CTSBp‐MUT (pcDNA3.4, pGL3‐Basic‐CTSBp‐MUT, pRL‐TK); STAT3+ CTSBp‐MUT, pcDNA3.4‐STAT3‐3×Myc, pGL3‐Basic‐CTSBp‐MUT, pRL‐TK). The luciferase activities of Renilla and Firefly were measured using the dual luciferase reporter assay kit (E1910, Promega, USA). The ratio of firefly luciferase to Renilla luciferase was defined as the relative luciferase activity.

### 2.21 Immunoprecipitation Assay

Regarding the binding between CTSB and S100A10, THP‐1‐polarized M0 macrophages added with IP lysis buffer (AWB0145, Abiowell, China) were sheared and lysed on ice for 30 min. The collected supernatants were divided into four groups: IgG‐input, IP‐input, IgG (B900610, Rabbit, Proteintech, China), S100A10‐IP (11250‐1‐AP, Rabbit, Proteintech, China). The collected supernatants were conjugated with Protein A/G Agarose Beads at 4°C for 2 h for immunoprecipitation. After that, the beads were washed with IP lysis buffer, and the eluted proteins were analyzed by western blot. CTSB (12216‐1‐AP, Rabbit, Proteintech, China) and S100A10 (11250‐1‐AP, Rabbit, Proteintech, China) were used as the primary antibodies. Goat anti‐Mouse IgG (H+L) (AWS0001, Abiowell, China) and Goat anti‐Rabbit IgG (H+L) (AWS0002, Abiowell, China) were used as the secondary antibodies.

Regarding the binding between CTSB and truncated S100A10, THP‐1‐polarized M0 macrophages after DNA transfection, added with IP lysis buffer (AWB0145, Abiowell, China), were sheared and lysed on ice for 30 min. The collected supernatants were conjugated with Protein A/G Agarose Beads at 4°C for 2 h for immunoprecipitation. After that, the beads were washed with IP lysis buffer, and the eluted proteins were analyzed by western blot. His (66005‐1‐Ig, Mouse, Proteintech, China) and Flag (66008‐4‐Ig, Rabbit, Proteintech, China) were used as the primary antibodies. Goat anti‐Mouse IgG (H+L) (AWS0001, Abiowell, China) and Goat anti‐Rabbit IgG (H+L) (AWS0002, Abiowell, China) were used as the secondary antibodies.

### 2.22 Molecular Docking

The crystal structures of CTSB (PDB ID: 8HE9) and S100A10 (PDB ID: 1A4P) were downloaded from the Protein Data Bank (http://www.rcsb.org/pdb/home/home.do). The Global Range Molecular Matching (GRAMM) software was used to predict the docking results and calculate the energy‐minimized optimal conformation.

### 2.23 RNA Sequencing Analysis

THP‐1 polarized M0 macrophages treated with or without human recombinant CTSB (rCTSB) protein (HY‐P78682, MCE, USA) were collected for RNA sequencing. The Illumina TruSeq Stranded Total RNA Library Prep Kit from Novogene was utilized to extract total RNA and build the library. After using Trim Galore to trim the raw FASTQ reads, HISAT2 [52] was used to map the transcripts to the human reference genome (hg38). The DEGs between the two groups were identified using the R package limma. The Metascape platform was employed for enrichment analysis of DEGs [53]. GO term and KEGG pathway enrichment analyses were also conducted for the DEGs.

### 2.24 Multiplex Immunofluorescence Staining

The human glioma tissue microarray (80 cores from 80 glioma samples) was purchased from the AiFang Biological company (AF‐BraSur2201, Changsha, China), and the ethics was approved. CTSB (Rabbit, 12216‐1‐AP, Proteintech, China), S100A10 (Rabbit, 11250‐1‐AP, Proteintech, China), CD68 (Rabbit, GB113150, Servicebio, China), CD163 (Rabbit, 16646‐1‐AP, Proteintech, China), were sequentially applied as primary antibodies, followed by horseradish peroxidase‐conjugated secondary antibody incubation (GB23301, GB23303, Servicebio, China) and tyramide signal amplification (TSA) (Servicebio, China). The detailed procedure was reported in the previous study [54]. TissueFAXS (TissueGnostics) was used to acquire the staining images. StrataQuest software (TissueGnostics) was used to quantify the number of total cells, the number of positive‐stained cells, the average intensity of positive‐stained cells, and the percentage of positive‐stained cells. The numbers of CD68^+^CD163^+^, S100A10^+^, CD68^+^S100A10^+^, and CD68^+^CD163^+^S100A10^+^ cells at different distances (0–25 µm, 25–50 µm, 50–100 µm, and 100–150 µm) from CTSB^+^ cells were quantified using StrataQuest software.

### 2.25 TAM Promotes CTSB‐mediated Malignancy in the GBM Subcutaneous Mouse Model

All animal experiments were conducted in strict compliance with institutional ethical guidelines and were approved by the Institutional Animal Care and Use Committee of The Second Affiliated Hospital, Chongqing Medical University. GL261 cells were subcutaneously injected into the right armpit of C57BL/6 male mice (six weeks, 5 × 10^5^ per mouse). The tumor volume was monitored every two days, three days after implantation. Five days after implantation, C57BL/6 male mice were randomly divided into two groups: NC and TAM. RAW 264.7 cells were polarized into TAMs after induction with IL‐4 (10 ng/mL). TAMs (5 × 10^5^ per mouse) were peritumorally injected every two days. All mice were sacrificed 13 days after implantation, and the tumor weight was monitored. HE staining was performed on the tumor tissues. Immunohistochemistry (IHC) staining of Ki67 (Primary antibody, AWA10320, Abiowell, China; Secondary antibody, AWI0629, Abiowell, China) was performed on the tumor tissues. Western blot was used to detect protein expression of CTSB, IL‐6, and STAT3. CTSB (12216‐1‐AP, Rabbit, Proteintech, China), IL‐6 (AWA42698, Rabbit, Abiowell, China), STAT3 (10253‐2‐AP, Rabbit, Proteintech, China), and β‐actin (66009‐1‐Ig, Mouse, Proteintech, China) were used as the primary antibodies. Goat anti‐Mouse IgG (H+L) (AWS0001, Abiowell, China) and Goat anti‐Rabbit IgG (H+L) (AWS0002, Abiowell, China) were used as the secondary antibodies.

### 2.26 TAM Promotes CTSB‐Mediated Malignancy in the GBM Orthotopic Mouse Model

All animal experiments were conducted in strict compliance with institutional ethical guidelines and were approved by the Institutional Animal Care and Use Committee of The Second Affiliated Hospital, Chongqing Medical University. Luc‐GL261 cells were intracranially injected into C57BL/6 male mice (six weeks, 1 × 10^5^ per mouse). Five days after implantation, C57BL/6 male mice were randomly divided into two groups: NC and TAM. RAW 264.7 cells were polarized into TAMs after induction with IL‐4 (10 ng/mL). TAMs (1 × 10^5^ per mouse) were peritumorally injected every two days. 13 days after implantation, C57BL/6 male mice were anaesthetized using isoflurane and intraperitoneally injected with D‐luciferin (150 mg/kg, AWHO736, Abiowell, China) to monitor tumor growth by bioluminescence imaging with the in vivo imaging system (IVIS Spectrum, Perkinelmer). All mice were sacrificed 15 days after implantation. HE staining was performed on the whole brain sections. IHC staining of Ki67 (Primary antibody, AWA10320, Abiowell, China; Secondary antibody, AWI0629, Abiowell, China) was performed on the tumor tissues. Western blot was used to detect protein expression of CTSB, IL‐6, and STAT3. CTSB (12216‐1‐AP, Rabbit, Proteintech, China), IL‐6 (AWA42698, Rabbit, Abiowell, China), STAT3 (10253‐2‐AP, Rabbit, Proteintech, China), and β‐actin (66009‐1‐Ig, Mouse, Proteintech, China) were used as the primary antibodies. Goat anti‐Mouse IgG (H+L) (AWS0001, Abiowell, China) and Goat anti‐Rabbit IgG (H+L) (AWS0002, Abiowell, China) were used as the secondary antibodies.

### 2.27 CTSB Mediates the Immunosuppressive Microenvironment in the GBM Subcutaneous Mouse Model

All animal experiments were conducted in strict compliance with institutional ethical guidelines and were approved by the Institutional Animal Care and Use Committee of The Second Affiliated Hospital, Chongqing Medical University. GL261 cells transfected with lentivirus shRNA‐NC and shRNA‐Ctsb were subcutaneously injected into the right armpit of C57BL/6 male mice (six weeks, 1 × 10^6^ per mouse). The tumor volume was monitored every two days, four days after implantation. All mice were sacrificed 12 days after implantation, and the tumor weight was monitored. Western blot was used to detect protein expression of CTSB. CTSB (12216‐1‐AP, Rabbit, Proteintech, China) was used as the primary antibody. Goat anti‐Mouse IgG (H+L) (AWS0001, Abiowell, China) and Goat anti‐Rabbit IgG (H+L) (AWS0002, Abiowell, China) were used as the secondary antibodies. Flow cytometry was used to detect CD45^+^, CD3^+^CD4^+^, CD3^+^CD8^+^, CD11b^+^CD86^+^, CD11b^+^CD206^+^, and PD‐L1^+^ cells in the tumor tissue. CD11b (APC, 17‐0112‐82, eBioscience, USA), CD45 (PC7, 25‐0452‐82, eBioscience, USA), CD86 (PE, 12‐0862‐82, eBioscience, USA), CD206 (PE, 12‐2061‐82, eBioscience, USA), CD3e (PC7, 25‐0032‐82, eBioscience, USA), CD4 (PE, 12‐0041‐82, eBioscience, USA), CD8a (APC, 17‐0081‐82, eBioscience, USA), and PD‐L1 (PE, 12‐5982‐82, eBioscience, USA) were used as the primary antibodies.

### 2.28 Knockdown of CTSB Combined With Anti‐PD‐1 Immunotherapy Has a Synergistic Effect in the GBM Subcutaneous Mouse Model

All animal experiments were conducted in strict compliance with institutional ethical guidelines and were approved by the Institutional Animal Care and Use Committee of The Second Affiliated Hospital, Chongqing Medical University. GL261 cells transfected with lentivirus shRNA‐NC and shRNA‐Ctsb were subcutaneously injected into the right armpit of C57BL/6 male mice (six weeks, 1 × 10^6^ per mouse). Four days after implantation, monoclonal antibody (10 mg/kg) (A2122, RMP1‐14, Selleck, USA) was injected via the tail vein every two days. C57BL/6 male mice were randomly divided into four groups: shRNA‐NC, shRNA‐Ctsb, shRNA‐NC+anti‐PD‐1 antibody, and shRNA‐Ctsb+anti‐PD‐1 antibody. The tumor volume was monitored every two days, four days after implantation. All mice were sacrificed 14 days after implantation, and the tumor weight was monitored. HE staining was performed on the tumor tissues. IHC staining of Ki67 (Primary antibody, AWA10320, Abiowell, China; Secondary antibody, AWI0629, Abiowell, China) was performed on the tumor tissues. TUNEL assay kit (40306ES50, YEASEN, China) was used to detect tumor cell apoptosis according to the manufacturer's protocol.

### 2.29 Knockdown of CTSB Combined With anti‐PD‐1 Immunotherapy Has a Synergistic Effect in the GBM Orthotopic Mouse Model

All animal experiments were conducted in strict compliance with institutional ethical guidelines and were approved by the Institutional Animal Care and Use Committee of The Second Affiliated Hospital, Chongqing Medical University. Luc‐GL261 cells or luc‐CT‐2A transfected with lentivirus shRNA‐NC and shRNA‐Ctsb were intracranially injected into C57BL/6 male mice (six weeks, 1 × 10^5^ per mouse). One week after implantation, anti‐PD‐1 monoclonal antibody (10 mg/kg) (A2122, RMP1‐14, Selleck, USA) was injected via the tail vein every two days. 16 days after implantation, C57BL/6 male mice were anaesthetized using isoflurane and intraperitoneally injected with D‐luciferin (150 mg/kg, AWHO736, Abiowell, China) to monitor tumor growth by bioluminescence imaging with the in vivo imaging system (IVIS Spectrum, Perkinelmer). All mice were sacrificed 18 days after implantation. HE staining was performed on the whole brain sections. IHC staining of Ki67 (Primary antibody, AWA10320, Abiowell, China; Secondary antibody, AWI0629, Abiowell, China) was performed on the tumor tissues. Western blot was used to detect protein expression of CTSB. CTSB (12216‐1‐AP, Rabbit, Proteintech, China) was used as the primary antibody. Goat anti‐Mouse IgG (H+L) (AWS0001, Abiowell, China) and Goat anti‐Rabbit IgG (H+L) (AWS0002, Abiowell, China) were used as the secondary antibodies. Flow cytometry was used to detect PD‐L1+ cells in the tumor tissue. PD‐L1 (PE, 12‐5982‐82, eBioscience, USA) was used as the primary antibody. Multiplex immunofluorescence (mIF) staining assay kit (AWI0693, Abiowell, China) was used to perform mIF staining of GFAP/CTSB, CD3/CD4, CD3/CD8, and CD11b/CD206 according to the manufacturer's protocol. CD11b (ab133357, Rabbit, Abcam, UK), CD206 (81525‐1‐RR, Rabbit, Proteintech, China), CTSB (ab270998, Rabbit, Abcam, UK), GFAP (ab7260, Rabbit, Abcam, UK), CD3 (17617‐1‐AP, Rabbit, Proteintech, China), CD4 (67786‐1‐Ig, Mouse, Proteintech, China), and CD8 (ab217344, Rabbit, Abcam, UK) were used as the primary antibodies. Ultrasensitive Goat anti‐Mouse/Rabbit IgG (AWI0629, Abiowell, China) was used as the secondary antibody.

### 2.30 Single‐cell RNA Sequencing Analysis on Orthotopic Tumor Tissues

The luc‐GL261 cells‐based orthotopic tumor tissues in the shRNA‐NC and shRNA‐Ctsb groups were collected for single‐cell RNA sequencing (scRNA‐seq) analysis. To create droplets using MobiNova‐100 (MobiDrop (Zhejiang) Co., Ltd., cat. no. A1A40001), dissociated cells were placed into a microfluidic chip of Chip A Single Cell Kit v2.1 (MobiDrop (Zhejiang) Co., Ltd., cat. no. S050100301). Every cell was included in a droplet that held a gel bead connected by millions of oligos (a barcode specific to each cell). Following encapsulation, oligos diffuse into the reaction mix while droplets are light‐cut by MobiNovaSP‐100 (MobiDrop (Zhejiang) Co., Ltd., cat. no. A2A40001). Cell barcodes using cDNA amplification in droplets were able to capture the mRNAs. After reverse transcription, barcoded cDNAs were amplified, and a library was created using the 3’ Dual Index Kit (MobiDrop (Zhejiang) Co., Ltd., cat. no. S050300301) and the High Throughput Single‐Cell 3’ Transcriptome Kit v2.1 (MobiDrop (Zhejiang) Co., Ltd., cat. no. S050200301) [55, 56]. An Illumina NovaSeq 6000 System was used to sequence the resultant libraries. The single‐cell 3’ transcriptome's raw data (fastq format) were pre‐analyzed using MobiVision (version 3.0, MobiDrop), and the reads were mapped to the Mus musculus reference GRCm39. Using MobiVision, a filtered cell‐gene matrix was produced for downstream analysis.

The R package Seurat was used to process the scRNA‐seq data of 7330 cells [57]. Uniform Manifold Approximation and Projection (UMAP) was used to present the distribution of cells. The InferCNV algorithm was employed to annotate single cancer cells at the single‐cell level. The R package CellChat was used to depict cell communication patterns [58]. DEGs between shRNA‐NC and shRNA‐Ctsb tumor cells were functionally annotated through GO term and KEGG pathway enrichment analyses.

### 2.31 Immunotherapy Response Prediction of CTSB

Immunotherapy cohorts were retrieved from ICBatlas [59] and ICBnetIS [49]. GBM immunotherapy dataset was derived from the cohort reported by Zhao [60]. For skin cutaneous melanoma (SKCM), immunotherapy‐treated patient samples were compiled across ten publicly available independent cohorts, namely Abril‐Rodriguez [61], Amato [62], Auslander [63], Freeman [64], Gide [65], Hugo [66], Liu [67], Riaz [68], Van‐Allen [69], and Zappasodi [70]. Additional non‐small cell lung cancer (NSCLC) immunotherapy cohorts from Cho [71] and Jung [72] were also integrated into the study.

### 2.32 Statistical Analysis

All statistical analyses and data visualization were performed using R software (version 4.4.0). The association between continuous variables was evaluated using Spearman's rank correlation coefficient. For group comparisons: Differences between two groups were assessed using the two‐tailed Student's t test; Differences among multiple groups were analyzed using the one‐way ANOVA test. All reported p‐values are two‐sided. To ensure result reliability, key experiments were repeated in multiple independent replicates to verify reproducibility.

## 3 Results

### 3.1 Identification of the TAM‐Related Genes by Biological Network Analyses

The distribution of the 19 TAM‐specific genes across human chromosomes is shown in Figure S1A. A PPIRWR procedure was performed to generate TAM‐associated gene ranking vectors (Figure 1A). GSEA of KEGG pathways was conducted via this ranking, revealing enrichment in pathways involved in macrophage activation and differentiation (Figure S1B). The TAM score was calculated by the ssGSEA method based on the 19 genes. A significant reduction in survival time was observed in GBM patients with high TAM scores (Figure S1C,D). WGCNA was performed to determine the TAM score‐related gene module from the top 10% of genes in the TAM‐associated PPIRWR‐derived gene ranking. The turquoise module exhibits the highest module‐trait correlation (correlation index = 0.79) with TAM score (Figure 1B). A high correlation (correlation index = 0.83) between module membership and gene significance for the TAM score was observed in the turquoise module (Figure 1C). Further, the WGCNA‐derived turquoise module was refined using MEGENA, resulting in a more condensed subnetwork. CTSB and FPR2 were identified as critical hub genes associated with TAM (Figure 1D).

**FIGURE 1:**
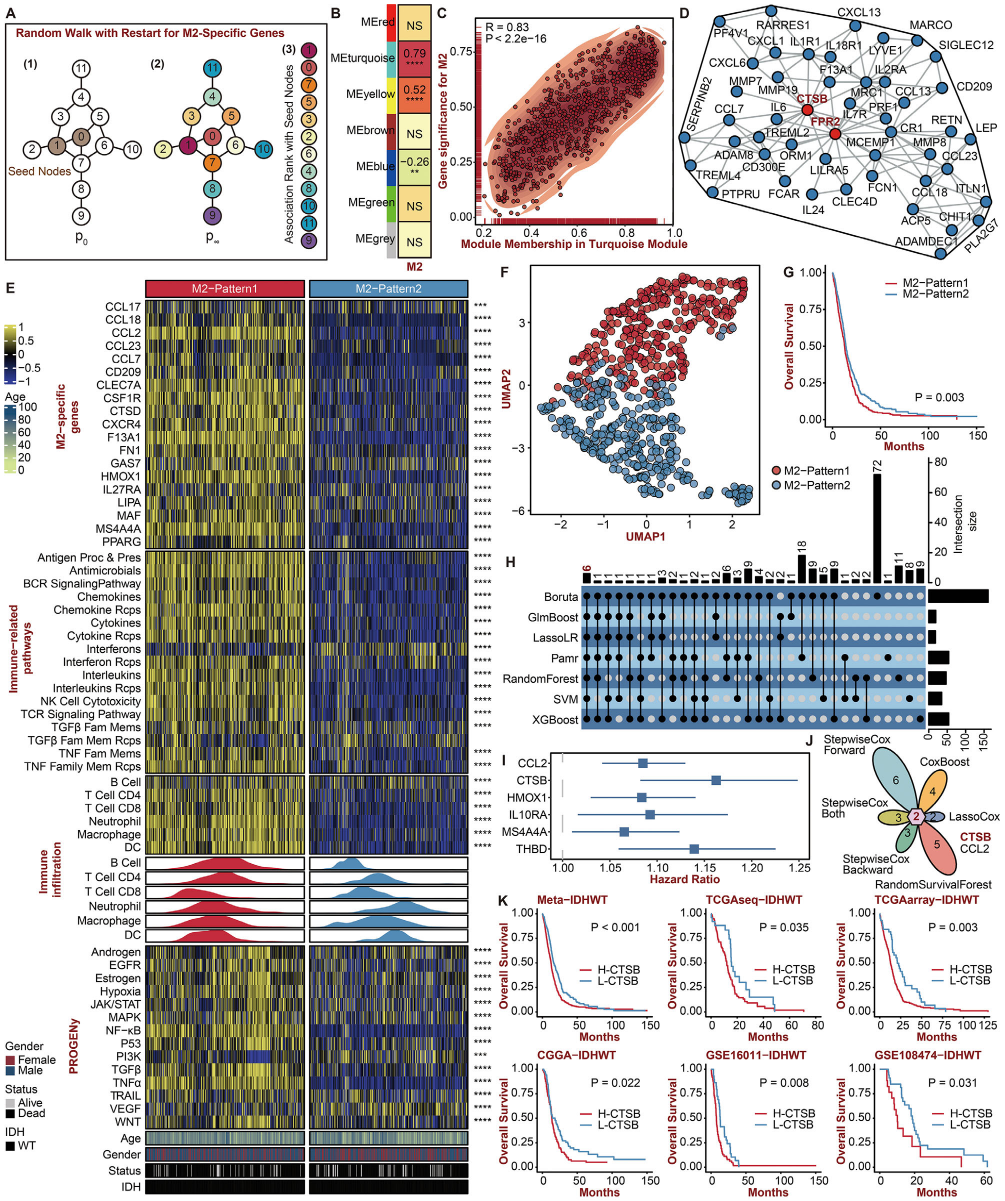
Identification of the TAM‐related Genes by Biological Network Analyses and Machine Learning A. PPIRWR procedure for generating TAM‐associated gene ranking vectors. B. WGCNA identified the turquoise module as the most correlated with the TAM score from the top 10% PPIRWR‐derived genes. C. The correlation between module membership and gene significance for the TAM score in the turquoise module. D. MEGENA further refined the WGCNA‐identified turquoise module into a more condensed subnetwork. E. The distribution of the TAM‐specific genes, ImmPort‐based immunological‐related pathways, TIMER‐based immune cell infiltration, and PROGENy‐based oncological‐related pathways in TAM‐pattern1 and TAM‐pattern2. F. UMAP analysis clearly distinguished two TAM‐patterns. G. Survival curves of two TAM‐patterns (log‐rank test). H. Seven ML algorithms for classification identified six intersecting genes crucial for TAM‐patterns. I. Univariate Cox regression analysis on six crucial genes. J. Six ML algorithms for survival pinpointed two genes as the most critical TAM‐related genes. K. Survival curves of CTSB‐based groups in six IDHWT GBM cohorts (log‐rank test).

### 3.2 Identification of the TAM‐Related Genes by Machine Learning

GBM patients were classified into two distinct groups (TAM‐pattern1 and TAM‐pattern2) using the PAM method based on the 19 TAM‐specific genes. GBM patients were divided into two patterns based on the 19 TAM‐specific genes, TAM‐pattern1 and TAM‐pattern2, using the PAM method. TAM‐pattern1 exhibited significantly higher levels of the TAM‐specific genes, ImmPort‐based immunological‐related pathways, TIMER‐based immune cell infiltration, and PROGENy‐based oncological‐related pathways (Figure 1E). UMAP analysis clearly distinguished these two patterns in GBM samples (Figure 1F). Importantly, patients with TAM‐pattern1 had worse survival outcomes (Figure 1G). Seven ML algorithms for classification, including Boruta, GlmBoost, LassoLR, Pamr, RF, SVM, and XGBoost, identified six intersecting genes crucial for TAM‐patterns (Figure 1H). All six genes were significantly associated with worse prognosis in GBM based on univariate Cox analysis (Figure 1I). Further, six ML algorithms for survival, including CoxBoost, LassoCox, StepwiseCoxForward, StepwiseCoxBoth, StepwiseCoxBackward, and RSF, pinpointed CTSB and CCL2 as the most critical TAM‐related genes (Figure 1J). Among these, CTSB emerged as particularly significant. This clinical importance was confirmed in six IDHWT GBM cohorts, where high CTSB expression consistently correlated with poorer survival (Figure 1K).

### 3.3 Functional Annotation and Immune Features of CTSB

The biological functions of CTSB were subsequently investigated. CTSB was found to be involved in T cell and macrophage activity, as revealed by GSEA of GO terms (Figure S2A). Through KEGG enrichment analysis, CTSB was closely associated with immunological and oncological pathways (Figure S2B). Furthermore, CTSB was identified as being highly involved in the cancer immune cycle (Figure S2C) and was correlated with cancer immunogram traits (Figure S2D). By using ESTIMATE, MCPcounter, Pornpimol‐ssGSEA, and TIMER algorithms, CTSB expression was observed to be linked to infiltration of immune cells, particularly macrophages, supporting its crucial role in the immunological environment (Figure S2E).

### 3.4 TAM Promotes CTSB‐mediated Malignancy

We first investigated the oncological roles of TAMs in GBM. TAMs were found to promote proliferation (Figure S3A) and migration (Figure S3B,C) of GBM cells. To explore the oncological roles of CTSB, the protein expression of CTSB was selectively silenced in GBM cells (Figure 2A,B). As expected, CTSB inhibition attenuated proliferation (Figure 2C) and migration (Figure 2D,E) in GBM cells. Next, to investigate the molecular linkage between CTSB‐mediated GBM progression and TAM activation, a co‐culture system was established for mechanistic validation. Interestingly, TAMs rescued the inhibited protein expression of CTSB (Figure 2F,G). Attenuated proliferation (Figure 2H) and migration (Figure 2I,J) in GBM cells with CTSB inhibition could be reversed by co‐culture with TAMs. Besides, CTSB inhibition promoted apoptosis in GBM cells, while subsequent co‐culture with TAMs suppressed apoptosis in GBM cells (Figure S4A–D). Notably, GBM cells with inhibited CTSB expression tended to be less arrested at S phase, while subsequent co‐culture with TAMs increased the proportion of GBM cells arrested at S phase (Figure S4E–H).

**FIGURE 2:**
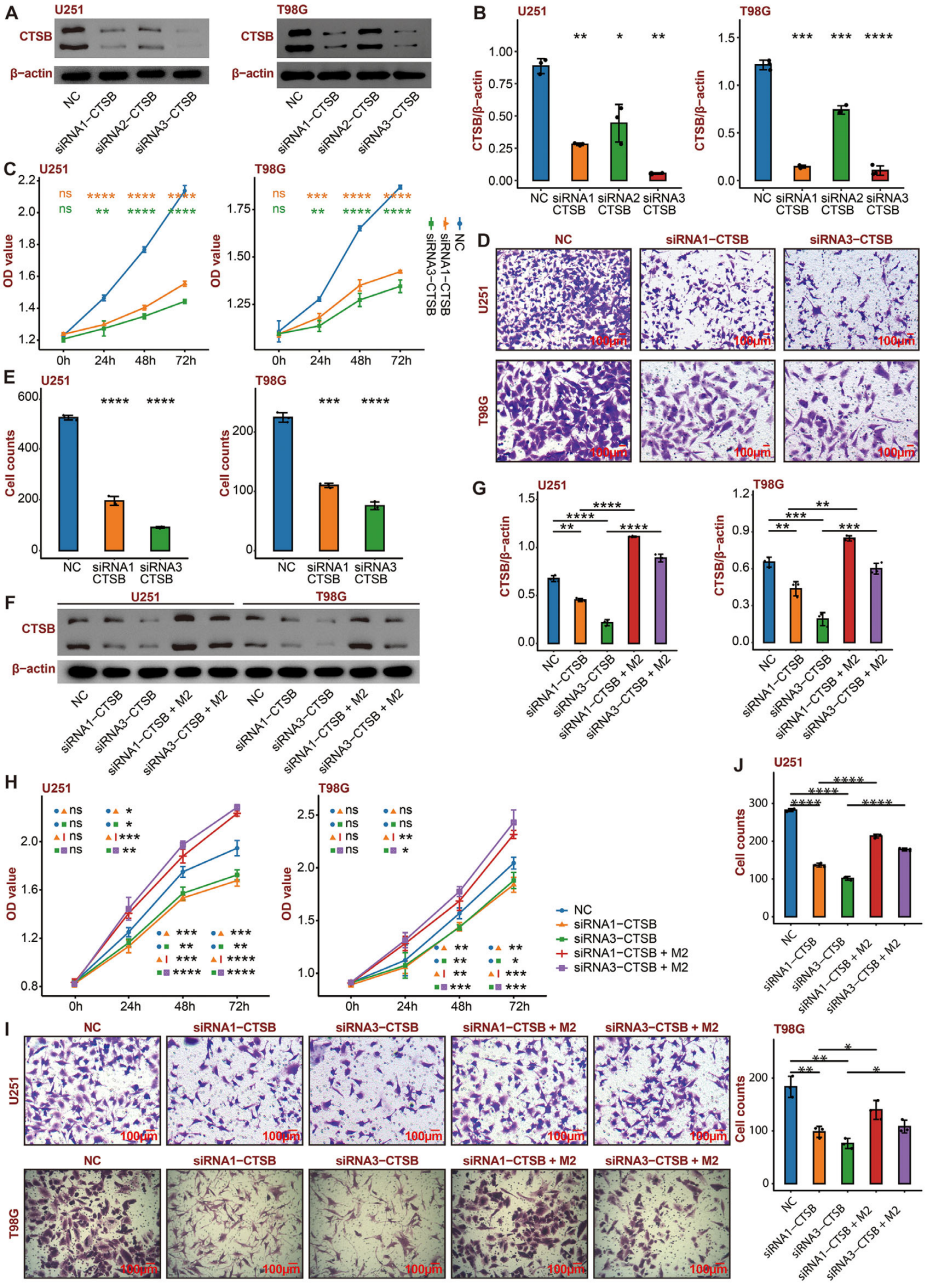
TAM Promotes CTSB‐mediated Malignancy A. Western blot shows the protein expression of CTSB in U251 and T98G cells transfected with siRNA‐NC and siRNA‐CTSB. B. The statistical results of the western blot assay for CTSB in U251 and T98G cells (n = 3, two‐tailed Student's t test). C. CCK8 shows the OD values of U251 and T98G cells transfected with siRNA‐NC and siRNA‐CTSB (n = 3, one‐way ANOVA test). D. Transwell shows the migrated U251 and T98G cells transfected with siRNA‐NC and siRNA‐CTSB. E. The statistical results of the Transwell assay for the migrated U251 and T98G cells (n = 3, one‐way ANOVA test). F. Western blot shows the protein expression of CTSB in U251 and T98G cells transfected with siRNA and co‐cultured with or without TAM. G. The statistical results of the western blot assay for CTSB (n = 3, one‐way ANOVA test). H. CCK8 shows the OD values of U251 and T98G cells transfected with siRNA and co‐cultured with or without TAM (n = 3, one‐way ANOVA test). I. Transwell shows the migrated U251 and T98G cells transfected with siRNA and co‐cultured with or without TAM. J. The statistical results of the Transwell assay for the migrated U251 and T98G cells (n = 3, one‐way ANOVA test). All data were presented as mean ± SD. *, *p* < 0.05; **, *p* < 0.01; ***, *p* < 0.001; ****, *p* < 0.0001; ns, not significant.

### 3.5 TAM Promotes CTSB‐Mediated Malignancy Via IL‐6/STAT3/CTSB Axis

The IL‐6 pathway was found to be closely linked with TAMs in GBM through GSEA analysis (Figure 3A). Thus, it was hypothesized that TAMs promoted CTSB‐mediated malignancy through IL‐6 signaling. The protein level of IL‐6 was significantly upregulated in the supernatant of GBM cells after co‐culture with TAMs (Figure 3B). Besides, immunofluorescence staining assay shows a more significant colocalization of IL‐6 and IL‐6R in the co‐culture group between GBM cells and TAMs (Figures S5A,B). Our study also revealed a significantly positive correlation between IL‐6 and STAT3 in GBM patients (Figure S5C). IL‐6/STAT3 axis has been widely reported to be hyperactivated in cancer [73], and STAT3 activation drives both tumor progression and immunosuppression in GBM [74]. Therefore, it was hypothesized that the IL‐6/STAT3 axis played a role in CTSB‐mediated malignancy.

**FIGURE 3:**
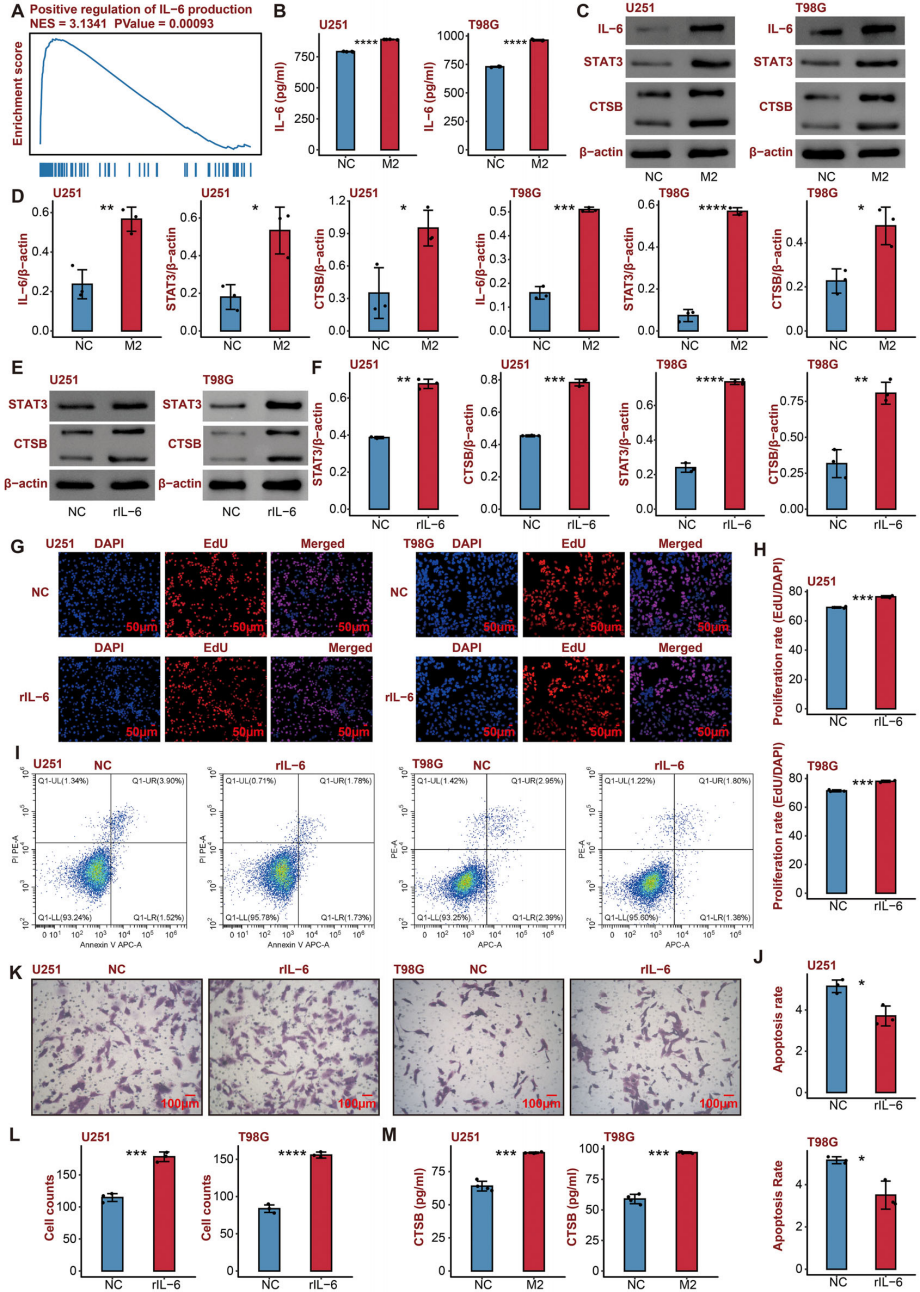
TAM Promotes CTSB‐mediated Malignancy via IL‐6/STAT3/CTSB Axis. A. The IL‐6 pathway was found to be closely linked with TAM in GBM through GSEA analysis. B. ELISA measures the protein level of IL‐6 in the supernatant of U251 and T98G cells after co‐culture with or without TAM (n = 3, two‐tailed Student's t test). C. Western blot shows the protein expression of IL‐6, STAT3, and CTSB in U251 and T98G cells after co‐culture with or without TAM. D. The statistical results of the western blot assay for IL‐6, STAT3, and CTSB (n = 3, two‐tailed Student's t test). E. Western blot shows the protein expression of STAT3 and CTSB in U251 and T98G cells after treatment with or without rIL‐6. F. The statistical results of the western blot assay for STAT3 and CTSB (n = 3, two‐tailed Student's t test). G. EdU shows the proliferated U251 and T98G cells after treatment with or without rIL‐6. H. The statistical results of the EdU assay for proliferated U251 and T98G cells (n = 3, two‐tailed Student's t test). I. Flow cytometry shows the proportion of apoptotic U251 and T98G cells after treatment with or without rIL‐6. J. The statistical results of the flow cytometry assay for apoptosis detection in U251 and T98G cells (n = 3, two‐tailed Student's t test). K. Transwell shows the migrated U251 and T98G cells after treatment with or without rIL‐6. L. The statistical results of the Transwell assay for the migrated U251 and T98G cells (n = 3, two‐tailed Student's t test). M. ELISA measures the protein level of CTSB in the supernatant of U251 and T98G cells in two groups (n = 3, two‐tailed Student's t test). All data were presented as mean ± SD. *, *p* < 0.05; **, *p* < 0.01; ***, *p* < 0.001; ****, *p* < 0.0001; ns, not significant.

As expected, IL‐6, STAT3, and CTSB protein expression were significantly elevated in GBM cells after co‐culture with TAMs (Figure 3C,D). Consistently, STAT3 and CTSB protein expression were significantly elevated in GBM cells after adding human recombinant IL‐6 protein (Figure 3E,F). Further, human recombinant IL‐6 protein promoted proliferation (Figure 3G,H), suppressed apoptosis (Figure 3I,J), and promoted migration (Figure 3K,L) in GBM cells. Conversely, STAT3 and CTSB protein expression were significantly reduced in GBM cells after adding IL‐6 monoclonal antibody (Figure S6A,B). IL‐6 monoclonal antibody suppressed proliferation (Figure S6C,D), promoted apoptosis (Figure S6E,F), and suppressed migration (Figure S6G,H) in GBM cells. The protein level of CTSB was also significantly upregulated in the supernatant of GBM cells after co‐culture with TAMs (Figure 3 M).

### 3.6 STAT3 Transcriptionally Activates CTSB and Promotes Malignancy in GBM

As STAT3 has been reported to be a classical transcription factor [75], it was hypothesized that STAT3 might directly regulate CTSB expression through transcriptional activation mechanisms. ChIP‐qPCR results demonstrated that STAT3 bound to the promoter region of CTSB in GBM cells (Figure 4A; Figure S7A). The optimal binding sequence and corresponding primer sequence are shown in Figure 4B. Dual‐luciferase reporter assay further verified that STAT3 significantly promoted the luciferase activity of CTSBp‐WT in comparison with CTSBp‐MUT in GBM cells (Figure 4C), confirming that STAT3 bound to the CTSB promoter and transcriptionally activated CTSB expression. Consistently, STAT3 overexpression rescued the inhibited protein expression of CTSB (Figure 4D,E). CTSB inhibition promoted apoptosis in GBM cells, while subsequent STAT3 overexpression suppressed apoptosis in GBM cells (Figure 4F,G). Besides, attenuated proliferation (Figures S7B,C) and migration (Figure 4H,I) in GBM cells with CTSB inhibition could be reversed by STAT3 overexpression.

**FIGURE 4:**
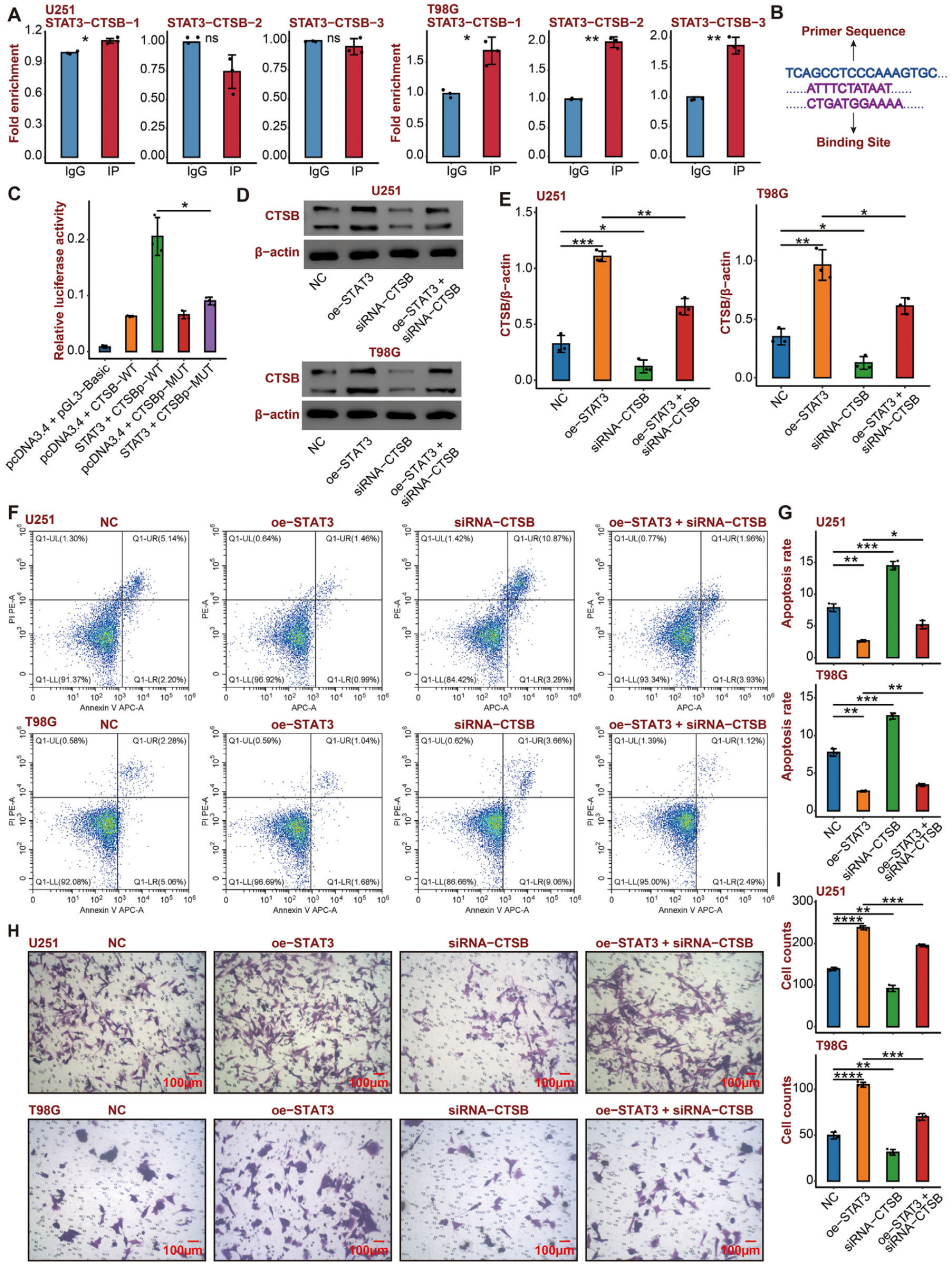
STAT3 Transcriptionally Activates CTSB and Promotes Malignancy in GBM. A. ChIP‐qPCR shows the STAT3 binding affinity to the different CTSB promoter regions in U251 and T98G cells (n = 3, two‐tailed Student's t test). B. The binding site of STAT3 on CTSB. C. Dual‐luciferase reporter assay shows the regulation of STAT3 in CTSB promoter activity in U251 cells (n = 3, one‐way ANOVA test). D. Western blot shows the protein expression of CTSB in U251 and T98G cells after transfection with STAT3‐overexpression plasmid and siRNA‐CTSB. E. The statistical results of the western blot assay for CTSB (n = 3, one‐way ANOVA test). F. Flow cytometry shows the proportion of apoptotic U251 and T98G cells after transfection with STAT3‐overexpression plasmid and siRNA‐CTSB. G. The statistical results of the flow cytometry assay for apoptosis detection in U251 and T98G cells (n = 3, one‐way ANOVA test). H. Transwell shows the migrated U251 and T98G cells in four groups. I. The statistical results of the Transwell assay for the migrated U251 and T98G cells (n = 3, one‐way ANOVA test). All data were presented as mean ± SD. *, *p* < 0.05; **, *p* < 0.01; ***, *p* < 0.001; ****, *p* < 0.0001; ns, not significant.

### 3.7 CTSB Promotes the Migration and Activation of TAM by Binding to the S100A10 Receptor

The activity of macrophages was further examined to assess the potential regulatory effects of GBM‐derived CTSB. Interestingly, inhibited activation of TAMs was observed after co‐culture with GBM cells with silenced CTSB expression (Figure 5A,B). Besides, inhibited migration of TAMs was observed after co‐culture with GBM cells with silenced CTSB expression (Figure 5C,D). ELISA analysis confirmed the secretion of CTSB by GBM cells, with significantly reduced CTSB levels observed in the supernatants of CTSB‐silenced U251 and T98G cells (Figure 5E). The predicted PPI network related to CTSB (http://www.interactome‐atlas.org/) is shown in Figure 5F. Notably, CTSB showed a strong correlation with S100A10 in GBM patients (Figure 5G). Additionally, Co‐IP assay showed the binding between CTSB and S100A10 in M0 macrophages (Figure 5H). Besides, immunofluorescence staining assay shows a more significant colocalization of CTSB and S100A10 in the co‐culture group between GBM cells and TAMs (Figure S8A,B). Previous findings have indicated that the C‐terminus of S100A10 is the critical domain for protein binding [76, 77]. Consistently, Co‐IP assay shows that CTSB had no interaction with S100A10 fragment (1‐79), indicating that the C‐terminus (80‐97) of S100A10 is required for the binding to CTSB (Figure 5I). Molecular docking shows the potential binding mode between CTSB and S100A10 by one chain comprising hydrophobic interactions, hydrogen bonds, π‐cation interactions, and salt bridges (Figure 5J). Notably, CTSB had potential binding sites on S100A10 at CYS‐82, TYR‐85, PHE‐86, MET‐90, and GLN‐92, within the C‐terminus (80‐97) region of S100A10. Subsequently, RNA sequencing analysis was performed on M0 macrophages after treatment with human recombinant CTSB protein. The DEGs between the two groups are shown in Figure S9A, among which IL‐6 had significantly higher expression in the rCTSB group (Figure S9B). ELISA detection further proved the inhibited protein level of IL‐6 in supernatant of TAMs after co‐culture with U251 and T98G cells with silenced CTSB expression (Figure 5K). Enrichment analysis demonstrated that tumor‐related pathways and immune‐related pathways, particularly those associated with macrophage effector functions, were significantly enriched in the rCTSB group (Figure S9C, D, E).

**FIGURE 5:**
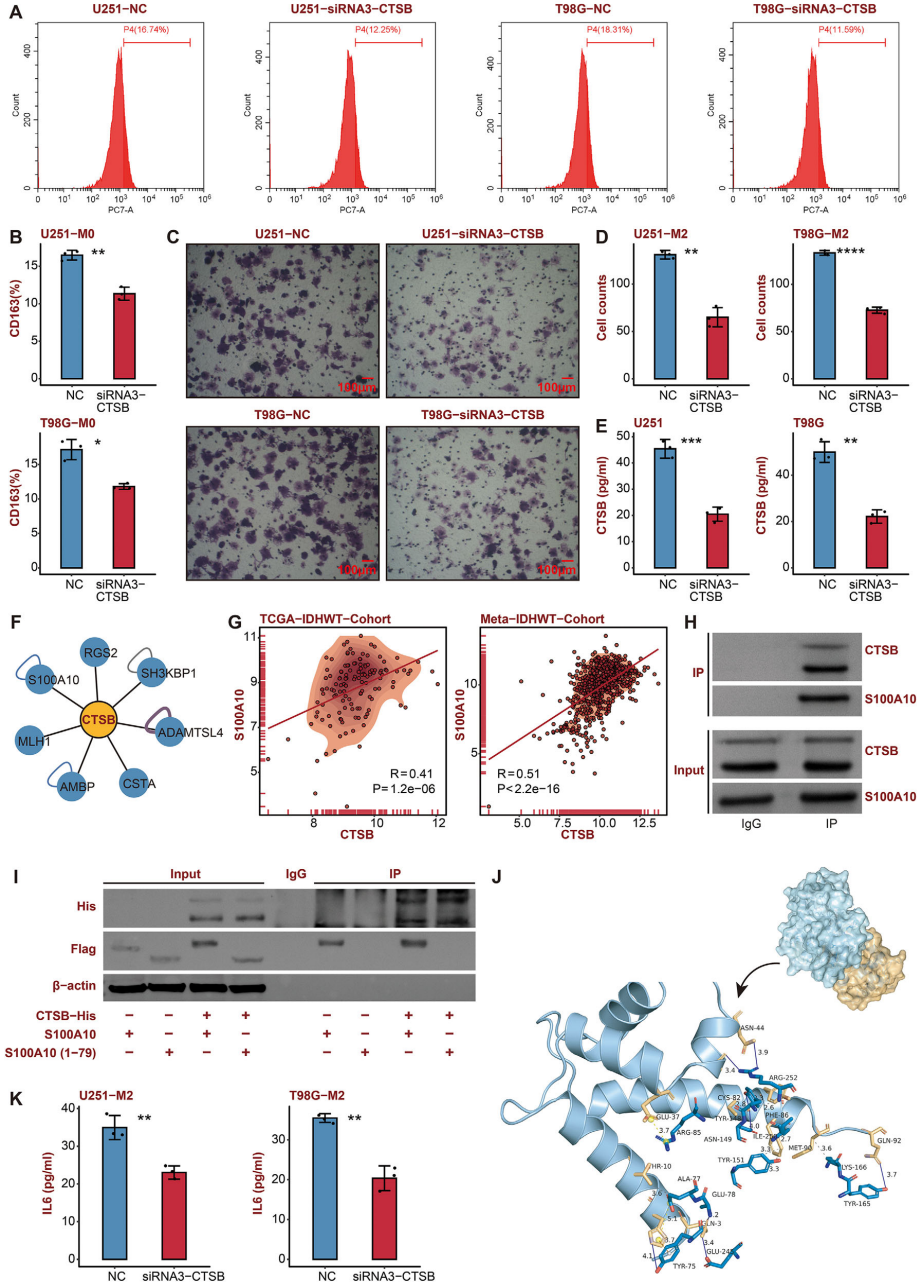
CTSB Promotes the Migration and TAM‐type Polarization of Macrophages by Binding to S100A10. A. Flow cytometry shows the proportion of CD163^+^ macrophages after co‐culture with U251 and T98G cells transfected with siRNA‐NC and siRNA‐CTSB. B. The statistical results of the flow cytometry assay for CD163^+^ macrophages after co‐culture with U251 and T98G cells, respectively (n = 3, two‐tailed Student's t test). C. Transwell shows the migrated TAM after co‐culture with U251 and T98G cells transfected with siRNA‐NC and siRNA‐CTSB. D. The statistical results of the Transwell assay for the migrated TAM after co‐culture with U251 and T98G cells, respectively (n = 3, two‐tailed Student's t test). E. ELISA measures the protein level of CTSB in the supernatant of U251 and T98G cells transfected with siRNA‐NC and siRNA‐CTSB (n = 3, two‐tailed Student's t test). F. The PPI network related to CTSB. G. The correlation between CTSB and S100A10 in the TCGA‐IDHWT GBM cohort and the meta‐IDHWT GBM cohort. H. Co‐IP assay confirmed the interaction the direct interaction between CTSB and S100A10 in M0 macrophages using anti‐S100A10 antibody. Immunoblotting showed the presence of CTSB in the IP lane. I. Co‐IP assay to detect the direct binding domain of CTSB on S100A10 using His‐CTSB and FLAG‐S100A10 truncation fragments (1‐79) in 293T cells. J. Molecular docking pattern between CTSB (Blue) and S100A10 (Yellow). K. ELISA measures the protein level of IL‐6 in the supernatant of TAM after co‐culture with U251 and T98G cells transfected with siRNA‐NC and siRNA‐CTSB (n = 3, two‐tailed Student's t test). All data were presented as mean ± SD. *, *p* < 0.05; **, *p* < 0.01; ***, *p* < 0.001; ****, *p* < 0.0001; ns, not significant.

To further explore how CTSB‐S100A10 protein interaction mediates macrophage activity, rescue experiments were designed. The RNA expression of S100A10 was significantly suppressed in M0 macrophages in three siRNA groups (Figure S10A). The inhibited protein level of IL‐6 in supernatants of M0 macrophages with S100A10 inhibition could be reversed by treating with the recombinant CTSB protein (Figure S10B). Attenuated migration in M0 macrophages with S100A10 inhibition could be reversed by treating with recombinant CTSB protein (Figure S10C,D). Besides, attenuated activation of TAMs with S100A10 inhibition could be reversed by treating with recombinant CTSB protein (Figure S10E,F). Multiplex immunofluorescence staining was performed to detect S100A10, CD68, CD163, and CTSB in glioma tissues. Representative images of multiplex immunofluorescence staining are displayed in Figures S11A, D, and G. CTSB^+^ cells were recognized by StrataQuest software (Figure S11B, E, H). Interestingly, CTSB^+^ cells were infiltrated by a higher density of CD68^+^CD163^+^, S100A10^+^, CD68^+^S100A10^+^, and CD68^+^CD163^+^S100A10^+^ cells, with greater infiltration observed at closer distances (Figure S11C, F, I). Notably, glioma patients with high CTSB^+^ cell counts (Figure S11J), S100A10^+^ cell counts (Figure S11K), CD68^+^S100A10^+^ cell counts (Figure S11L), and CD68^+^CD163^+^S100A10^+^ cell counts (Figure S11M) had relatively decreased survival time.

### 3.8 TAM Promotes CTSB‐mediated Malignancy in the GBM Subcutaneous and Orthotopic Mouse Models

Subsequently, the potential role of TAMs in promoting CTSB‐mediated malignancy was further explored using a GBM subcutaneous mouse model. The resected subcutaneous tumor tissues in the two groups are shown in Figure 6A. The group with injection of TAMs had significantly higher tumor volume (Figure 6B) and tumor weight (Figure 6C). The HE staining further confirmed more atypical nuclei in the TAMs group (Figure 6D). Moreover, TAMs significantly promoted tumor proliferation, as demonstrated by IHC staining of Ki67 (Figure 6E,F). Besides, TAMs significantly promoted IL‐6, STAT3, and CTSB protein expression in subcutaneous tumor tissues, consistent with the previous findings (Figure 6G).

**FIGURE 6:**
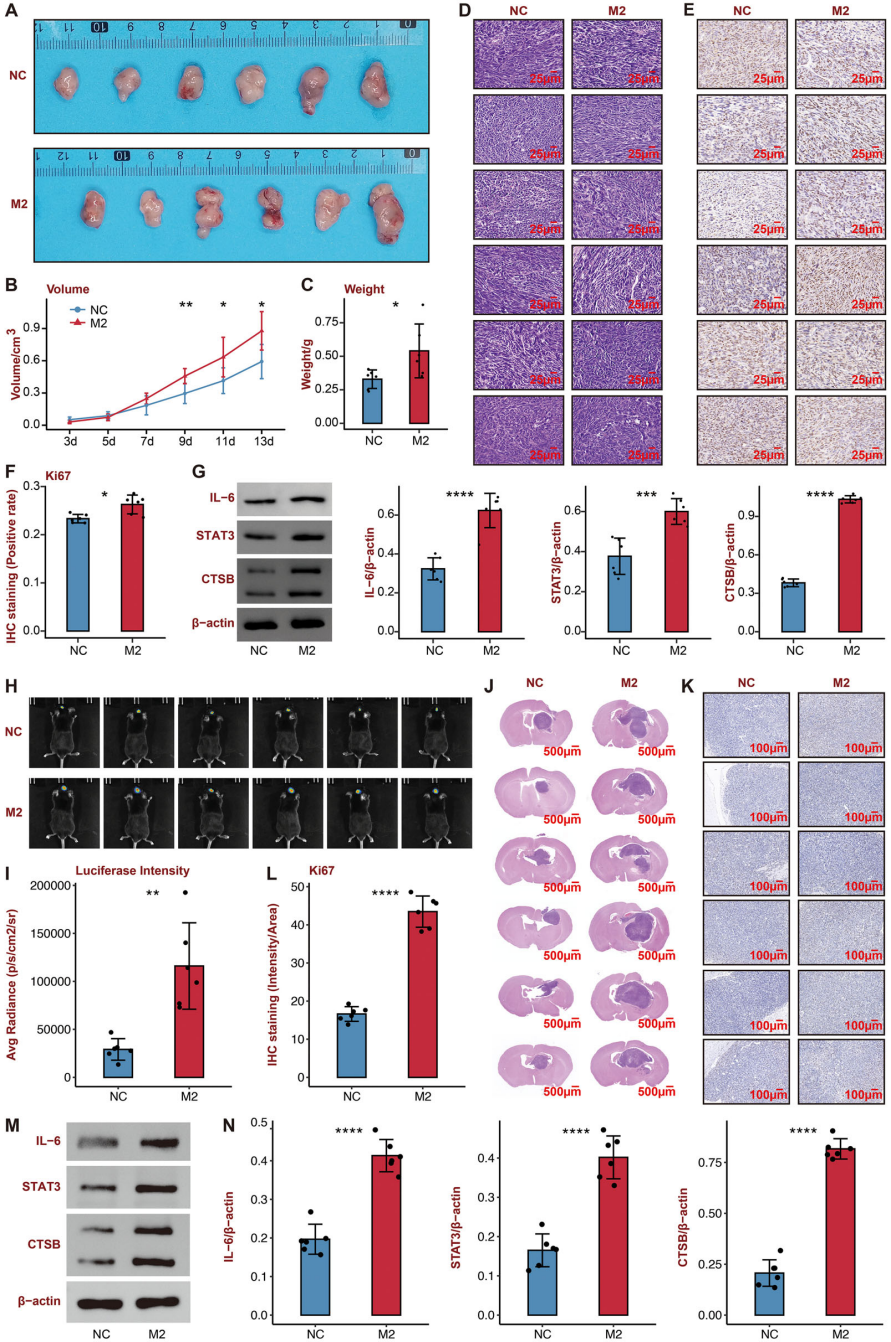
TAM Promotes CTSB‐mediated Malignancy in the GBM Subcutaneous and Orthotopic Mouse Models. A. The images of subcutaneous tumor tissues with or without injection of TAM. B. The tumor volume at different time points in subcutaneous tumor tissues with or without injection of TAM (n = 6, two‐tailed Student's t test). C. The tumor weight in subcutaneous tumor tissues with or without injection of TAM (n = 6, two‐tailed Student's t test). D. The HE staining images of subcutaneous tumor tissues with or without injection of TAM. E. The IHC staining images of Ki67 in subcutaneous tumor tissues with or without injection of TAM. F. The statistical results of the IHC staining assay for Ki67 (n = 6, two‐tailed Student's t test). G. Western blot shows the protein expression of IL‐6, STAT3, and CTSB in subcutaneous tumor tissues with or without injection of TAM. The statistical results of the western blot assay for IL‐6, STAT3, and CTSB (n = 6, two‐tailed Student's t test). H. In vivo bioluminescence images of orthotopic tumor tissues with or without injection of TAM. I. The statistical analysis of bioluminescent tracking plots. J. The HE staining images of orthotopic tumor tissues with or without injection of TAM. K. The IHC staining images of Ki67 in orthotopic tumor tissues with or without injection of TAM. L. The statistical results of the IHC staining assay for Ki67 (n = 6, two‐tailed Student's t test). M. Western blot shows the protein expression of IL‐6, STAT3, and CTSB in orthotopic tumor tissues with or without injection of TAM. N. The statistical results of the western blot assay for IL‐6, STAT3, and CTSB (n = 6, two‐tailed Student's t test).

The protumor effects of TAMs observed in the subcutaneous model were further validated in the orthotopic GBM model. In vivo bioluminescence imaging (Figure 6H,I) and HE staining (Figure 6J) confirmed enhanced tumor growth and increased nuclear atypia in the TAMs group. Consistent with the subcutaneous model, TAMs significantly elevated Ki67 positivity (Figure 6K,L) and upregulated IL‐6, STAT3, and CTSB protein expression (Figure 6 M,N).

### 3.9 CTSB Mediates the Immunosuppressive Microenvironment in the GBM Subcutaneous Mouse Model

The role of CTSB in mediating the tumor microenvironment was further explored in the GBM subcutaneous mouse model. The resected subcutaneous tumor tissues are shown in Figure S13A. CTSB protein expression was significantly inhibited in the shRNA‐Ctsb group (Figure S13B). The shRNA‐Ctsb group had significantly lower tumor volume (Figure S13C) and tumor weight (Figure S13D). Notably, the proportion of CD11b^+^CD86^+^ macrophages was significantly higher in the shRNA‐Ctsb group (Figure S13E,I), while CD11b^+^CD206^+^ macrophages were significantly lower in the shRNA‐Ctsb group (Figure S13F,J). Besides, the proportion of CD3^+^CD4^+^ T cells (Figure S13G,K) and CD3^+^CD8^+^ T cells (Figure S13H,L) was significantly higher in the shRNA‐Ctsb group. Taken together, these results indicate that CTSB promotes activation of TAMs and suppresses infiltration of T cells.

### 3.10 Knockdown of CTSB Combined With anti‐PD‐1 Immunotherapy Has a Synergistic Effect in the GBM Subcutaneous Mouse Model

Given that T cells [78] and macrophages [79] are both pivotal determinants of cancer immunotherapy, the impact of CTSB on immunotherapy efficacy in GBM was investigated. TAMs were positively associated with PD‐L1 in GBM patients (Figure S12A). Notably, CTSB was shown to interact with immune checkpoints (Figure S12B) and align with immunotherapeutic signatures (Figure S12C), highlighting its potential as an effective immunotherapeutic biomarker. CTSB silencing led to reduced protein expression of PD‐L1 in GBM cells (Figure 7A,B). Consistently, the proportion of PD‐L1^+^ cells was significantly lower in the shRNA‐Ctsb group in the GBM subcutaneous mouse model (Figure 7C,D). Thus, it was hypothesized that targeting CTSB could enhance the therapeutic efficacy of anti‐PD‐1 immunotherapy. As expected, targeting CTSB augmented the efficacy of anti‐PD‐1 immunotherapy in the GBM subcutaneous mouse model (Figure 7E), as the shRNA‐Ctsb combined with anti‐PD‐1 antibody group had significantly lower tumor volume (Figure 7F) and tumor weight (Figure 7G) than the shRNA‐Ctsb group and the anti‐PD‐1 antibody group, respectively. The HE staining further confirmed fewer atypical nucleus in the shRNA‐Ctsb combined with anti‐PD‐1 antibody group (Figure 7H). Tumor proliferation was more restrained in the shRNA‐Ctsb combined with anti‐PD‐1 antibody group, as demonstrated by IHC staining of Ki67 (Figure 7I,K). Moreover, the proportion of apoptotic cells was significantly higher in the shRNA‐Ctsb combined with anti‐PD‐1 antibody group, as demonstrated by TUNEL staining (Figure 7J,L).

**FIGURE 7:**
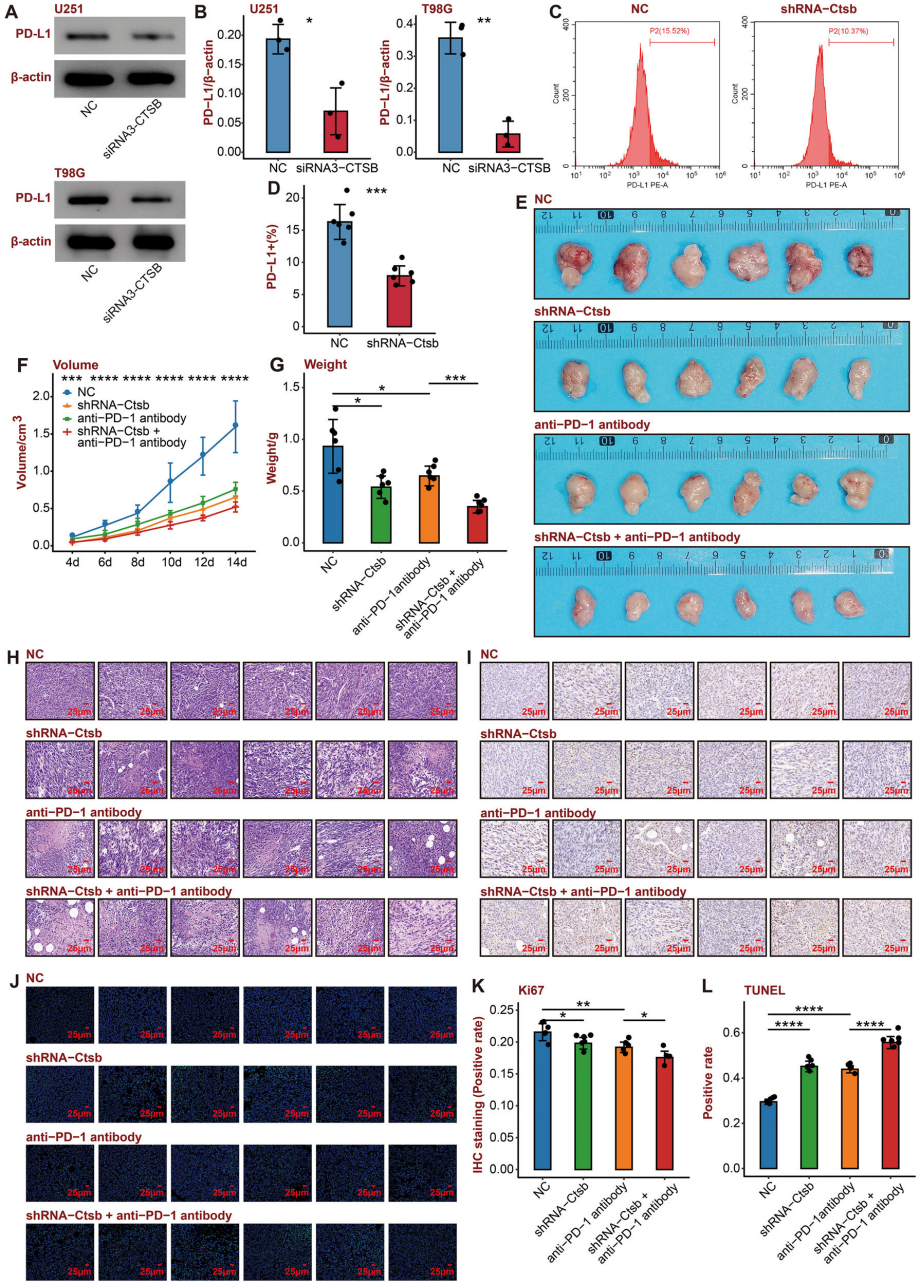
Knockdown of CTSB Combined With anti‐PD‐1 Immunotherapy Has a Synergistic Effect in the GBM Subcutaneous Mouse Model. A. Western blot shows the protein expression of PD‐L1 in U251 and T98G cells transfected with siRNA‐NC and siRNA‐CTSB. B. The statistical results of the western blot assay for PD‐L1 (n = 3, two‐tailed Student's t test). C. Flow cytometry shows the proportion of PD‐L1^+^ cells in subcutaneous tumor tissues with or without CTSB knockdown. D. The statistical results of the flow cytometry assay for PD‐L1^+^ cells (n = 6, two‐tailed Student's t test). E. The images of subcutaneous tumor tissues under different treatments. F. The tumor volume at different time points in subcutaneous tumor tissues under different treatments (n = 6, one‐way ANOVA test). G. The tumor weight in subcutaneous tumor tissues under different treatments (n = 6, one‐way ANOVA test). H. The HE staining images of subcutaneous tumor tissues under different treatments. I. The IHC staining images of Ki67 in subcutaneous tumor tissues under different treatments. J. The TUNEL staining images of subcutaneous tumor tissues under different treatments. K. The statistical results of the IHC staining assay for Ki67 (n = 6, one‐way ANOVA test). L. The statistical results of the TUNEL staining assay (n = 6, one‐way ANOVA test). All data were presented as mean ± SD. *, *p* < 0.05; **, *p* < 0.01; ***, *p* < 0.001; ****, *p* < 0.0001; ns, not significant.

### 3.11 Knockdown of CTSB Combined With anti‐PD‐1 Immunotherapy Has a Synergistic Effect in the GBM Orthotopic Mouse Model

The synergistic effect of targeting CTSB combined with anti‐PD‐1 immunotherapy was verified in the GL261 GBM orthotopic mouse model. CTSB protein expression was significantly inhibited in the shRNA‐Ctsb group (Figure 8A). As expected, the proportion of PD‐L1^+^ cells was significantly lower in the shRNA‐Ctsb group in the GBM orthotopic mouse model (Figure 8B). In vivo bioluminescence images revealed the most restrained orthotopic tumor growth in the shRNA‐Ctsb combined with anti‐PD‐1 antibody group (Figure 8C,D). The IHC staining (Figure 8E,F) and HE staining (Figure 8G) images further confirmed the most restrained orthotopic tumor growth in the shRNA‐Ctsb combined with anti‐PD‐1 antibody group. Besides, the proportion of CD11b^+^CD206^+^ macrophages was significantly lower in the shRNA‐Ctsb group, while the proportion of CD3^+^CD4^+^ T cells and CD3^+^CD8^+^ T cells were significantly higher in the shRNA‐Ctsb group (Figure 8H,I), confirming that CTSB promotes activation of TAMs and suppresses infiltration of T cells. Notably, anti‐PD‐1 immunotherapy prolonged mice survival time, and targeting CTSB could improve the therapeutic efficacy of anti‐PD‐1 immunotherapy (Figure 8J).

**FIGURE 8:**
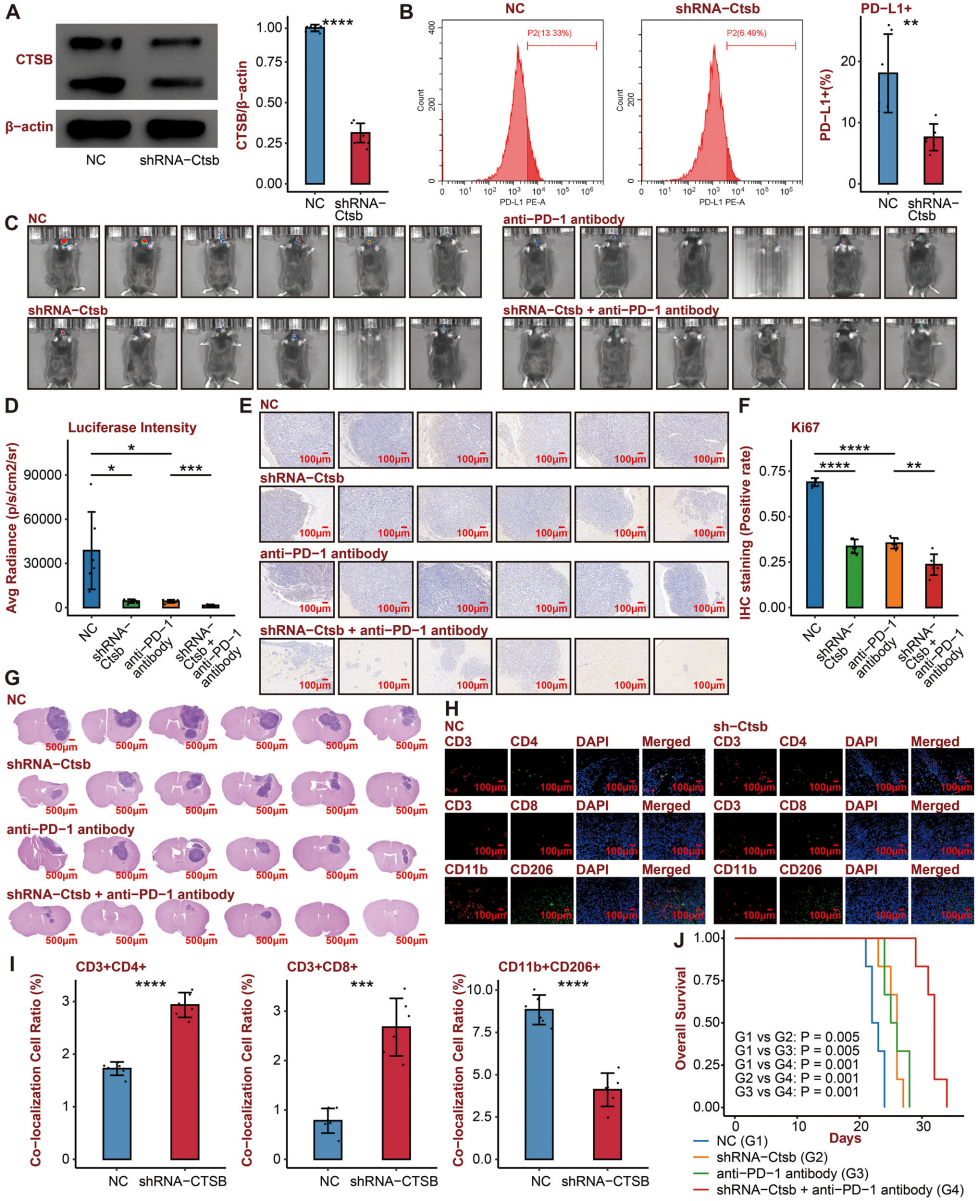
Knockdown of CTSB Combined With anti‐PD‐1 Immunotherapy Has a Synergistic Effect in the GL261 GBM Orthotopic Mouse Model. A. Western blot shows the protein expression of CTSB in orthotopic tumor tissues with or without CTSB knockdown. The statistical results of the western blot assay for CTSB (n = 6, two‐tailed Student's t test). B. Flow cytometry shows the proportion of PD‐L1^+^ cells in orthotopic tumor tissues with or without CTSB knockdown. The statistical results of the flow cytometry assay for PD‐L1^+^ cells (n = 6, two‐tailed Student's t test). C. In vivo bioluminescence images of orthotopic tumor tissues under different treatments. D. The statistical analysis of bioluminescent tracking plots (n = 6, one‐way ANOVA test). E. The IHC staining images of Ki67 in orthotopic tumor tissues under different treatments. F. The statistical results of the IHC staining assay for Ki67 (n = 6, one‐way ANOVA test). G. The HE staining images of orthotopic tumor tissues under different treatments. H. Multiplex immunofluorescence staining of CTSB/GFAP, CD3/CD4, CD3/CD8, and CD11b/CD206 in orthotopic tumor tissues with or without CTSB knockdown. I. The statistical results of the co‐localization ratio of CD3^+^CD4^+^ cells, CD3^+^CD8^+^ cells, and CD11b^+^CD206^+^ cells in the two groups (n = 6, two‐tailed Student's t test). J. Survival curves of C57BL/6 mice under different treatments (log‐rank test). All data were presented as mean ± SD. *, *p* < 0.05; **, *p* < 0.01; ***, *p* < 0.001; ****, *p* < 0.0001; ns, not significant.

The consistent synergistic effect of combining CTSB targeting with anti‐PD‐1 immunotherapy was further confirmed in the CT‐2A GBM orthotopic model. This independent validation recapitulated the key findings: successful CTSB knockdown (Figure 9A) was associated with reduced PD‐L1 expression (Figure 9B) and a significant reduction in tumor burden in the combination group, as visualized by bioluminescence imaging (Figure 9C,D) and confirmed by IHC (Figure 9E,F) and H&E staining (Figure 9G). The immunomodulatory role of CTSB knockdown was also reiterated, characterized by a lower proportion of TAMs (CD11b^+^CD206^+^) and enhanced T cell infiltration (CD3^+^CD4^+^, CD3^+^CD8^+^) (Figure 9H,I). Ultimately, the survival advantage conferred by the combination therapy in this model (Figure 9J) reinforced the robustness of this therapeutic strategy.

**FIGURE 9:**
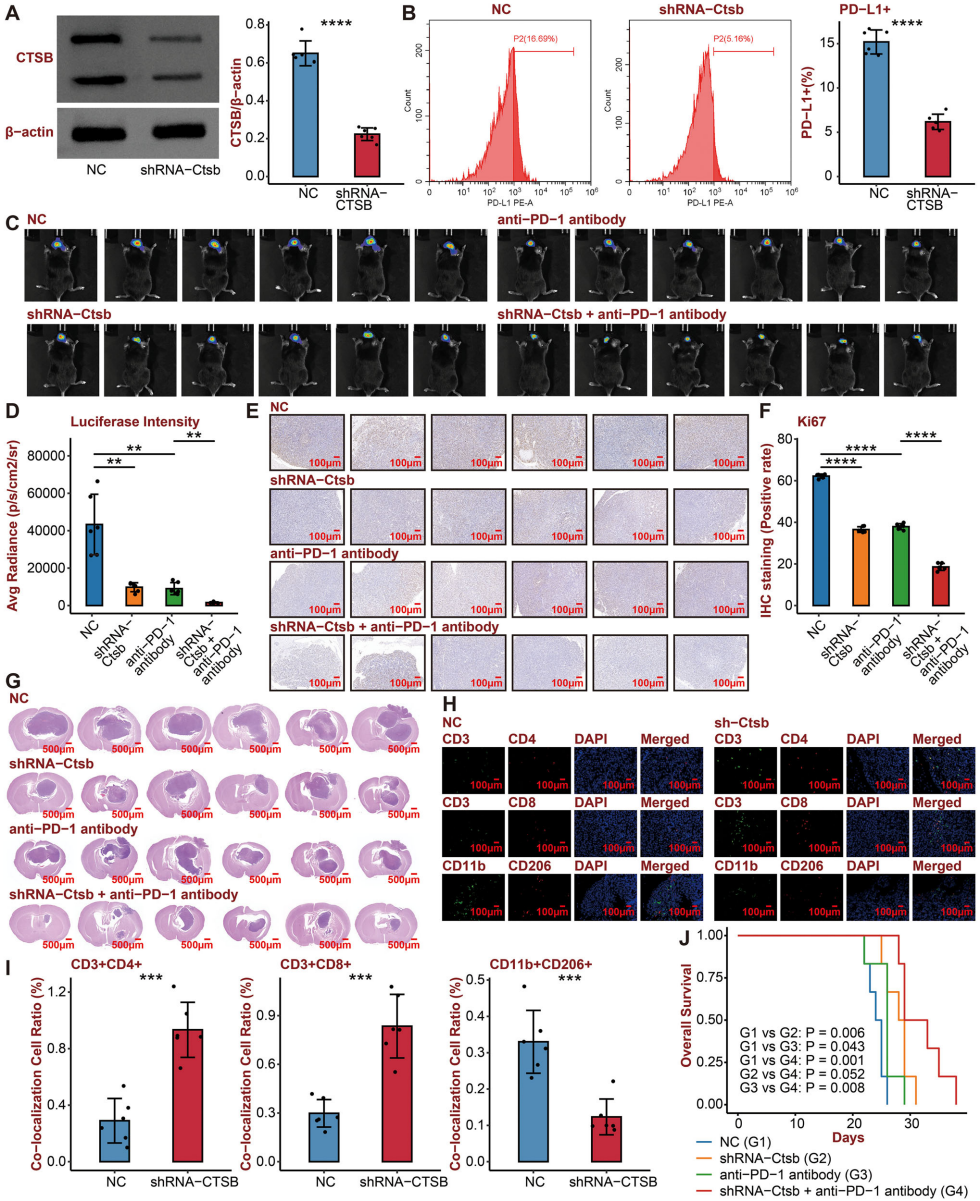
Knockdown of CTSB Combined With anti‐PD‐1 Immunotherapy Has a Synergistic Effect in the CT‐2A GBM Orthotopic Mouse Model. A. Western blot shows the protein expression of CTSB in orthotopic tumor tissues with or without CTSB knockdown. The statistical results of the western blot assay for CTSB (n = 6, two‐tailed Student's t test). B. Flow cytometry shows the proportion of PD‐L1^+^ cells in orthotopic tumor tissues with or without CTSB knockdown. The statistical results of the flow cytometry assay for PD‐L1^+^ cells (n = 6, two‐tailed Student's t test). C. In vivo bioluminescence images of orthotopic tumor tissues under different treatments. D. The statistical analysis of bioluminescent tracking plots (n = 6, one‐way ANOVA test). E. The IHC staining images of Ki67 in orthotopic tumor tissues under different treatments. F. The statistical results of the IHC staining assay for Ki67 (n = 6, one‐way ANOVA test). G. The HE staining images of orthotopic tumor tissues under different treatments. H. Multiplex immunofluorescence staining of CTSB/GFAP, CD3/CD4, CD3/CD8, and CD11b/CD206 in orthotopic tumor tissues with or without CTSB knockdown. I. The statistical results of the co‐localization ratio of CD3^+^CD4^+^ cells, CD3^+^CD8^+^ cells, and CD11b^+^CD206^+^ cells in the two groups (n = 6, two‐tailed Student's t test). J. Survival curves of C57BL/6 mice under different treatments (log‐rank test). All data were presented as mean ± SD. *, *p* < 0.05; **, *p* < 0.01; ***, *p* < 0.001; ****, *p* < 0.0001; ns, not significant.

Corroborating our in vivo validation data, we next interrogated the clinical association between CTSB expression and immunotherapeutic response across pan‐cancer immunotherapy cohorts. Across GBM, SKCM, and NSCLC immunotherapy cohorts, CTSB expression was consistently and significantly downregulated in immunotherapy responders relative to non‐responders (Figure S14). These clinical findings mechanistically align with our preceding functional data demonstrating that CTSB silencing augments anti‐PD‐1 therapeutic efficacy. Collectively, our cohort analyses establish that reduced CTSB abundance correlates with favorable immunotherapeutic outcomes, further supporting the translational potential of CTSB as an actionable target to enhance clinical immunotherapy response.

Besides, scRNA‐seq analysis was performed on orthotopic tumor tissues in the shRNA‐NC and shRNA‐Ctsb groups. The integrated samples in the two groups are shown in Figure 10A. Seven major microenvironment cell types, including macrophages, monocytes, natural killer (NK) cells, T cells, endothelial cells, pericytes, and oligodendrocytes, were identified (Figure 10B). Further, M0/M1/M2 macrophages and cancer cells were identified (Figure 10C). Notably, TAMs took up a lower percentage while T cells took up a higher percentage in the tumor microenvironment of the shRNA‐Ctsb group (Figure 10D). The cell‐cell interaction network revealed that cancer cells interacted less with TAMs in the shRNA‐Ctsb group (Figure 10E). Enrichment analysis on DEGs between the shRNA‐NC and shRNA‐Ctsb groups revealed that macrophage activity‐related pathways were enriched (Figure 10F).

**FIGURE 10:**
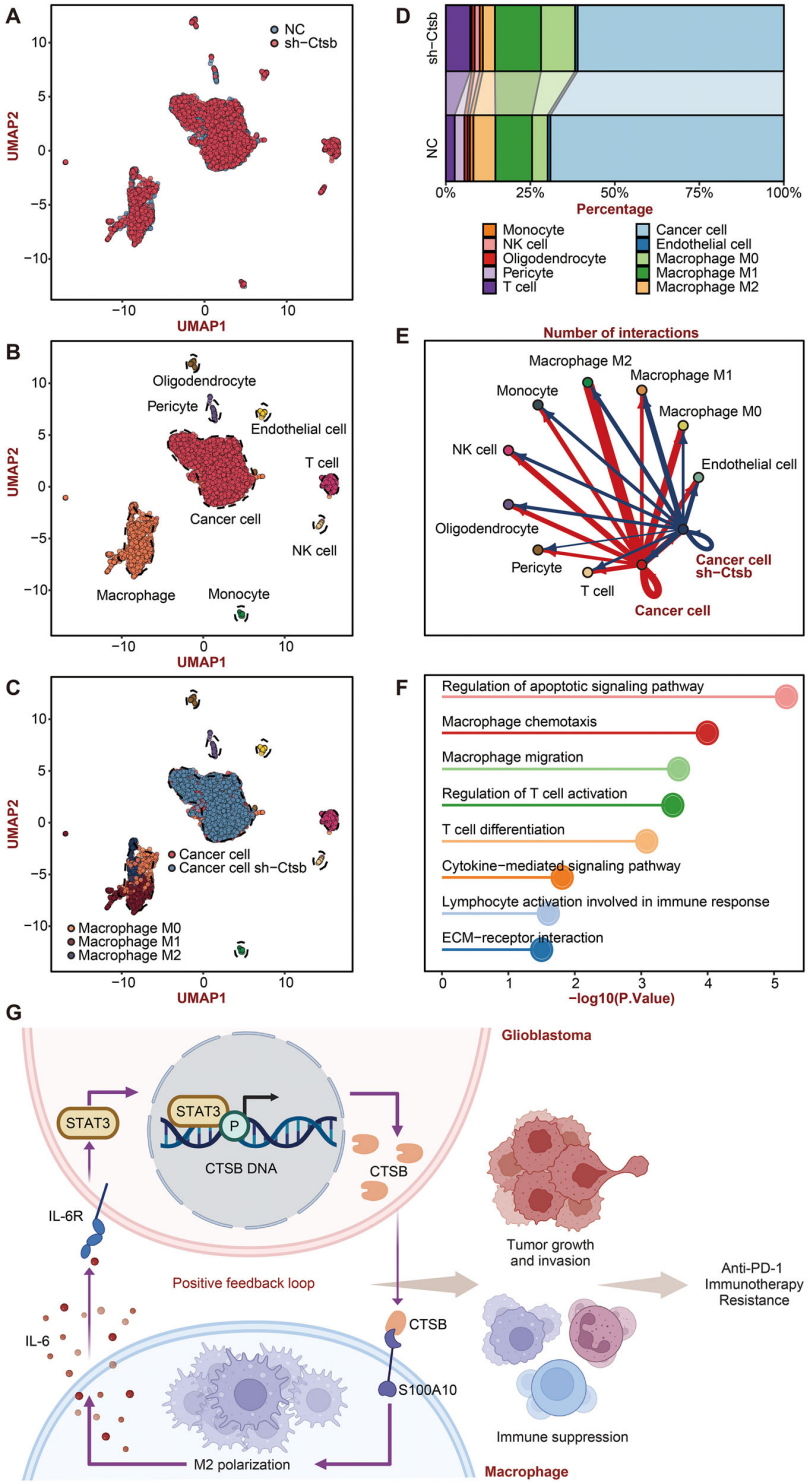
The scRNA‐seq Analysis on Orthotopic Tumor Tissues. A. UMAP shows the integration of samples in the two groups (shRNA‐NC and shRNA‐Ctsb). B. Seven major microenvironment cell types were identified. C. M0/M1/M2 macrophages and cancer cells were further identified. D. The percentage of microenvironment cells in the two groups. E. Cell‐cell interaction network among microenvironment cells. F. Enrichment analysis based on the DEGs between shRNA‐NC and shRNA‐Ctsb tumor cells. G. A working model showing that TAMs secrete IL‐6 to activate STAT3 in GBM cells, which transcriptionally upregulates CTSB expression. Secreted CTSB binds S100A10 on macrophages, reinforcing their M2 polarization and reciprocal IL‐6 production, establishing a feedforward IL‐6/STAT3/CTSB/S100A10 axis. Functionally, this loop enhances GBM proliferation, invasion, and immunotherapy resistance. All data were presented as mean ± SD. *, *p* < 0.05; **, *p* < 0.01; ***, *p* < 0.001; ****, *p* < 0.0001; ns, not significant.

## 4 Discussion

GBM remains a formidable therapeutic challenge due to its immunosuppressive microenvironment and resistance to immunotherapy. In this study, we uncovered a previously unrecognized feedforward circuit centered on the direct molecular dialogue between CTSB^+^ GBM cells and S100A10^+^ macrophages, which drives tumor progression and immune evasion. Through multi‐dimensional analyses of large‐scale IDH‐wildtype GBM samples along with experimental validation, CTSB emerged as a key regulator of this immunosuppressive network, offering new therapeutic targets. These findings not only reveal a critical tumor‐macrophage crosstalk mechanism but also demonstrate how integrative computational and experimental approaches can dissect complex tumor‐microenvironment interactions (Figure 10G).

Artificial intelligence has emerged as a transformative tool for identifying biomarkers predictive of cancer prognosis and therapeutic sensitivity [10, 11, 12]. In the current study, biological network analyses revealed CTSB as a topologically pivotal gene within the TAM‐associated gene module. ML algorithms further highlighted CTSB's centrality as a molecular hub governing TAM function. Clinical data and in vitro experiments further established CTSB as a hazardous gene in GBM, reinforcing the value of integrative computational approaches in target discovery. CTSB, a lysosomal cysteine protease, has pleiotropic roles in extracellular matrix degradation and cytokine processing [80]. However, its regulation by tumor‐TAM interactions and immunomodulatory functions in GBM remains unknown. To prove that CTSB is a pivotal mediator of GBM‐TAM crosstalk, the current findings integrated mechanistic and functional evidence. While IL‐6/STAT3 signaling is well‐documented in tumor immunosuppression [73, 81], the uniqueness of our work lies in elucidating how CTSB, transcriptionally regulated within tumor cells, functions extracellularly to engage S100A10 on macrophages. S100A10, traditionally studied in the context of plasminogen recruitment and macrophage invasion [82, 83], emerges here as a functional receptor for tumor‐derived CTSB. It was noteworthy that the C‐terminus of S100A10 was responsible for tumor invasion [77] and macrophage invasion [82]. The predicted binding sites of CTSB on S100A10 at CYS‐82, TYR‐85, PHE‐86, MET‐90, and GLN‐92 were likely to be potential targets. This dynamic bidirectional circuit establishes a self‐sustaining tumor microenvironment, wherein progressive immunosuppression and invasive tumor growth are mutually reinforced. Notably, spatial multiplex imaging of human glioma tissues revealed that CTSB^+^ cells were closely adjacent to S100A10^+^ TAMs, and such spatial organization highlights the clinical relevance of this axis.

The therapeutic implications of targeting this loop were underscored by preclinical models (GL261 and CT‐2A), where CTSB knockdown reversed TAM activation, reinvigorated CD8^+^ T cell infiltration, and synergized with anti‐PD‐1 therapy to suppress tumor growth. Notably, the synergy between CTSB inhibition and PD‐1 blockade exceeded additive effects, suggesting immune contexture remodeling rather than mere pathway blockade. The convergence of these key findings in both models is particularly telling. These results align with emerging evidence that disrupting tumor‐microenvironment communication can potentiate checkpoint blockade [84]. For instance, cancer cell‐intrinsic CD28 promotes immune escape in triple‐negative breast cancer by stabilizing PD‐L1 mRNA, and targeting CD28 enhances anti‐tumor immunity and improves anti‐PD‐1 efficacy [85]. Notably, CTSB inhibition not only reduced PD‐L1 expression but also reshaped the myeloid compartment, shifting macrophages toward an immunostimulatory phenotype. This noteworthy effect, which reduced immunosuppressive cues, positioned CTSB as a pivotal node for combination therapies. The scRNA‐seq data generated in this study further supported this, revealing diminished TAM interactions and enriched anti‐tumor immune pathways in CTSB‐deficient tumors.

In summary, by integrating multi‐dimensional omics data, orthogonal functional validation across in vitro and in vivo models, and spatial confirmation in human tissues, this study defines a targetable immunosuppressive axis centered on CTSB^+^ GBM cells and S100A10^+^ macrophages. Disrupting this dialogue not only curbs tumor progression but also reprograms the immune microenvironment to sensitize GBM to immunotherapy. From a translational perspective, CTSB serves as a promising therapeutic target to reverse immune suppression and enhance anti‑PD‑1 immunotherapy. Future investigations will focus on developing and optimizing brain‑penetrant, highly selective CTSB inhibitors with ideal pharmacokinetic properties for intracranial tumors. Combination regimens of CTSB inhibitors plus anti‑PD‑1/PD‑L1 therapy warrant clinical evaluation, especially in patients with high CTSB expression and poor immunotherapy response. Our findings thus provide a solid mechanistic and translational rationale for targeting CTSB to improve outcomes in GBM patients.

However, certain limitations should be noted. First, while our spatial analysis confirms proximity between CTSB^+^ and S100A10^+^ cells, further investigations using higher‐resolution spatial transcriptomics or proteomics could delineate the communication dynamics within distinct tumor niches. Second, the precise downstream signaling events following CTSB–S100A10 engagement warrant deeper exploration—for example, whether S100A10 dimerization, membrane recruitment, or co‐receptor involvement modulates macrophage reprogramming. Finally, although we used two orthotopic GBM models, additional patient‐derived xenograft or immunocompetent humanized models could better capture the heterogeneity of human GBM–macrophage interactions.

## Author Contributions

Quan Cheng, Hao Zhang and Nan Zhang conceived the study. Hao Zhang and Nan Zhang drafted the manuscript. Hao Zhang, Wantao Wu, Xisong Liang, and Jie Wen conducted the experiments. Nan Zhang, Hao Zhang, and Ziyu Dai performed the computational analyses. Quan Cheng, Wantao Wu, and Hao Zhang provided funding support. Peng Luo, Quan Cheng, Hao Zhang, Nan Zhang, Zhiwei Xia, Songshan Feng, Wantao Wu, Shuyu Li, Xisong Liang, Jie Wen, Ziyu Dai, and Zaoqu Liu revised the manuscript. All authors read and approved the final manuscript.

## Funding

This work was supported by the National Natural Science Foundation of China (NO. 82372943, 82303610, 82503442), Chongqing Medical Youth Top Talent Program (NO. YXQN202558), Chongqing Youth Innovation Talent Project (NO. CSTB2025YITP‐QCRCX0096), The Science and Technology Innovation Program of Hunan Province (NO. 2023RC3074), Key R&D Program of Jiangxi Province (NO. 20243BBI91007), Jiangxi Provincial Health Technology Project (NO. 202510008), Beijing Xisike Clinical Oncology Research Foundation (NO. Y‐Gilead2024‐PT‐0070), Jiangxi Provincial Natural Science Foundation (NO. 20252BAC220048).

## Ethics Statement

All animal experiments were conducted strictly with institutional ethical guidelines and were approved by the Institutional Animal Care and Use Committee of The Second Affiliated Hospital, Chongqing Medical University (Approval NO. IACUC‐SAHCQMU‐2023‐0017). The human glioma tissue microarray (80 cores from 80 glioma samples) was purchased from the AiFang Biological company (AF‐BraSur2201, Changsha, China), and the ethical approval was obtained.

## Consent for Publication

Written informed consent was obtained from all patients.

## Conflicts of Interest

The authors declare no conflicts of interest.

## Supporting information




**Supporting File 1**: advs76905‐sup‐0001‐SuppMat.docx.

**Supporting File 2**: advs76905‐sup‐0002‐SuppTableS1‐S4.xlsx.

## Data Availability

The data that support the findings of this study are openly available in TCGA (The Cancer Genome Atlas), CGGA (Chinese Glioma Genome Atlas), and GEO (Gene Expression Omnibus). The sequencing data generated during this study has been submitted to the National Genomics Data Center and are available from the corresponding author upon reasonable request.
