# Supplementary material for: Crosstalk Between CTSB+ Glioblastoma Cells and S100A10+ Macrophages: A Self‐Reinforcing Circuit Promotes Immune Evasion and Limits Response to Immunotherapy
Source: Adv Sci (Weinh). 2026 Aug 29;13(59):e76905. doi: 10.1002/advs.76905 (PMC13525416; doi:10.1002/advs.76905)
Supplement: Supplementary file 1 [file ADVS-13-e76905-s002.docx]

**Supplementary Figures**

**Figure S1. Identification of the TAM-related genes by biological network analyses.**

A. The distribution of the 19 TAM-specific genes across human chromosomes in the TCGA-IDHWT GBM cohort. B. GSEA based on the TAM-associated PPIRWR-derived gene ranking in the TCGA-IDHWT GBM cohort. C. Survival curves of TAM score-based groups in the meta-IDHWT GBM cohort (log-rank test). D. Survival curves of TAM score-based groups in the TCGA-IDHWT GBM cohort (log-rank test).

**Figure S2. Functional annotation and immune features of CTSB.**

A. GSEA-based immunological GO terms related to CTSB in the TCGA-IDHWT GBM cohort. B. KEGG-based immunological and oncological pathways related to CTSB in the TCGA-IDHWT GBM cohort. C. The correlation between CTSB and the cancer immune cycle in the TCGA-IDHWT GBM cohort. D. The correlation between CTSB and cancer immunogram traits in the TCGA-IDHWT GBM cohort. E. The correlation between CTSB and infiltration of immune cells evaluated by ESTIMATE, MCPcounter, Pornpimol-ssGSEA, and TIMER algorithms in the TCGA-IDHWT GBM cohort.

**Figure S3. TAMs promote GBM malignancy.**

A. CCK8 shows the OD values of U251 and T98G cells co-cultured with or without TAMs (n = 3, two-tailed Student’s t test). B. Transwell shows the migrated U251 and T98G cells co-cultured with or without TAMs. C. The statistical results of the Transwell assay for the migrated U251 and T98G cells (n = 3, two-tailed Student’s t test). All data were presented as mean ± SD. *, P < 0.05; **, P < 0.01; ***, P < 0.001; ****, P < 0.0001; ns, not significant.

**Figure S4. TAM promotes CTSB-mediated malignancy.**

A. Flow cytometry shows the proportion of apoptotic cells in U251 cells in five groups. B. Flow cytometry shows the proportion of apoptotic cells in T98G cells in five groups. C. The statistical results of the flow cytometry assay for apoptosis detection in U251 cells (n = 3, one-way ANOVA test). D. The statistical results of the flow cytometry assay for apoptosis detection in T98G cells (n = 3, one-way ANOVA test). E. Flow cytometry shows the proportion of U251 cells at different cell cycles. F. Flow cytometry shows the proportion of T98G cells at different cell cycles. G. The statistical results of the flow cytometry assay for cell cycle detection in U251 cells (n = 3, one-way ANOVA test). H. The statistical results of the flow cytometry assay for cell cycle detection in T98G cells (n = 3, one-way ANOVA test). All data were presented as mean ± SD. *, P < 0.05; **, P < 0.01; ***, P < 0.001; ****, P < 0.0001; ns, not significant.

**Figure S5. TAM-derived IL-6 binds to IL-6R receptor on GBM cells.** A. Immunofluorescence staining of IL-6 and IL-6R in U251 and T98G cells after co-culture with or without TAMs. B. The statistical results of immunofluorescence staining assay for proportion of IL-6^+^IL-6R^+^ positive cells (n = 3, two-tailed Student’s t test). C. The correlation between IL-6 and STAT3 in the TCGA-IDHWT GBM cohort and the meta-IDHWT GBM cohort. All data were presented as mean ± SD. *, P < 0.05; **, P < 0.01; ***, P < 0.001; ****, P < 0.0001; ns, not significant.

**Figure S6. TAM promotes CTSB-mediated malignancy via IL-6/STAT3/CTSB axis.**

A. Western blot assay shows the protein expression of STAT3 and CTSB in U251 and T98G cells in two groups. B. The statistical results of the western blot assay for STAT3 and CTSB (n = 3, two-tailed Student’s t test). C. EdU shows the proliferated U251 and T98G cells in two groups. D. The statistical results of the EdU assay for proliferated U251 and T98G cells (n = 3, two-tailed Student’s t test). E. Flow cytometry shows the proportion of apoptotic U251 and T98G cells in the two groups. F. The statistical results of the flow cytometry assay for apoptosis detection in U251 and T98G cells (n = 3, two-tailed Student’s t test). G. Transwell shows the migrated U251 and T98G cells in two groups. H. The statistical results of the Transwell assay for the migrated U251 and T98G cells (n = 3, two-tailed Student’s t test). All data were presented as mean ± SD. *, P < 0.05; **, P < 0.01; ***, P < 0.001; ****, P < 0.0001; ns, not significant.

**Figure S7. TAMs promote CTSB-mediated malignancy via IL-6/STAT3/CTSB axis.**

A. ChIP-qPCR shows the STAT3 binding affinity to the different CTSB promoter regions in LN229 cells. B. EdU shows the proliferated U251 and T98G cells after transfection with STAT3-overexpression plasmid and siRNA-CTSB. C. The statistical results of EdU assay for proliferated U251 and T98G cells (n = 3, one-way ANOVA test). All data were presented as mean ± SD. *, P < 0.05; **, P < 0.01; ***, P < 0.001; ****, P < 0.0001; ns, not significant.

**Figure S8. GBM-derived CTSB binds to S100A10 receptor on TAMs.** A. Immunofluorescence staining of CTSB and S100A10 in TAMs after co-culture with or without U251 and T98G cells. B. The statistical results of immunofluorescence staining assay for proportion of CTSB^+^S100A10^+^ positive cells (n = 3, two-tailed Student’s t test). All data were presented as mean ± SD. *, P < 0.05; **, P < 0.01; ***, P < 0.001; ****, P < 0.0001; ns, not significant.

**Figure S9. RNA sequencing analysis on THP-1 polarized M0 macrophages treated with or without human recombinant CTSB protein.**

A. Volcano plot displays the DEGs between two groups. B. The expression level of IL-6 in the two groups. C. Enrichment analysis based on the DEGs by Metascape. D. GSEA analysis of GO and KEGG based on the DEGs. E. Enrichment analysis of GO and KEGG based on the DEGs.

**Figure S10. CTSB promotes the migration and TAM-type polarization of macrophages by binding to the S100A10 receptor.**

A. RT-qPCR shows the RNA expression of S100A10 in M0 macrophages in four groups (n = 3, one-way ANOVA test). B. ELISA measures the protein level of IL-6 in the supernatant of M0 macrophages in four groups (n = 3, one-way ANOVA test). C. Transwell shows the migrated M0 macrophages in four groups. D. The statistical results of the Transwell assay for the migrated M0 macrophages (n = 3, one-way ANOVA test). E. Flow cytometry shows the proportion of CD68^+^CD163^+^ macrophages in four groups. F. The statistical results of the flow cytometry assay for detection of CD68^+^CD163^+^ macrophages (n = 3, one-way ANOVA test). All data were presented as mean ± SD. *, P < 0.05; **, P < 0.01; ***, P < 0.001; ****, P < 0.0001; ns, not significant.

**Figure S11. Multiplex immunofluorescence staining of S100A10, CD68, CD163, and CTSB in glioma tissues.**

A. Representative images of multiplex immunofluorescence staining. B. CTSB^+^ cells recognized by StrataQuest software. C. The number of CD68^+^CD163^+^, S100A10^+^, CD68^+^S100A10^+^, and CD68^+^CD163^+^S100A10^+^ cells at different distances from CTSB^+^ cells. D. Representative images of multiplex immunofluorescence staining. E. CTSB^+^ cells recognized by StrataQuest software. F. The number of CD68^+^CD163^+^, S100A10^+^, CD68^+^S100A10^+^, and CD68^+^CD163^+^S100A10^+^ cells at different distances from CTSB^+^ cells. G. Representative images of multiplex immunofluorescence staining. H. CTSB^+^ cells recognized by StrataQuest software. I. The number of CD68^+^CD163^+^, S100A10^+^, CD68^+^S100A10^+^, and CD68^+^CD163^+^S100A10^+^ cells at different distances from CTSB^+^ cells. J. Survival curves of CTSB^+^ cell count-based groups in glioma patients (log-rank test). K. Survival curves of S100A10^+^ cell count-based groups in glioma patients (log-rank test). L. Survival curves of CD68^+^S100A10^+^ cell count-based groups in glioma patients (log-rank test). M. Survival curves of CD68^+^CD163^+^S100A10^+^ cell count-based groups in glioma patients (log-rank test).

**Figure S12. The predictive value of CTSB in immunotherapy.**

A. The correlation between TAMs and PD-L1 in the TCGA-IDHWT GBM cohort. B. The correlation between CTSB and PD-1, PD-L1, and CTLA-4 in the TCGA-IDHWT GBM cohort. C. The correlation between CTSB and ten immunotherapeutic signatures in the TCGA-IDHWT GBM cohort.

**Figure S13. CTSB mediates the immunosuppressive microenvironment in the GBM subcutaneous mouse model.**

A. The images of subcutaneous tumor tissues with or without CTSB knockdown. B. Western blot shows the protein expression of CTSB in subcutaneous tumor tissues with or without CTSB knockdown. The statistical results of the western blot assay for CTSB (n = 6, two-tailed Student’s t test). C. The tumor volume at different time points in subcutaneous tumor tissues with or without CTSB knockdown (n = 6, two-tailed Student’s t test). D. The tumor weight in subcutaneous tumor tissues with or without CTSB knockdown (n = 6, two-tailed Student’s t test). E. Flow cytometry shows the proportion of CD11b^+^CD86^+^ macrophages in subcutaneous tumor tissues with or without CTSB knockdown. F. Flow cytometry shows the proportion of CD11b^+^CD206^+^ macrophages in subcutaneous tumor tissues with or without CTSB knockdown. G. Flow cytometry shows the proportion of CD3^+^CD4^+^ T cells in subcutaneous tumor tissues with or without CTSB knockdown. H. Flow cytometry shows the proportion of CD3^+^CD8^+^ T cells in subcutaneous tumor tissues with or without CTSB knockdown. I. The statistical results of the flow cytometry assay for CD11b^+^CD86^+^ macrophages (n = 6, two-tailed Student’s t test). J. The statistical results of the flow cytometry assay for CD11b^+^CD206^+^ macrophages (n = 6, two-tailed Student’s t test). K. The statistical results of the flow cytometry assay for CD3^+^CD4^+^ T cells (n = 6, two-tailed Student’s t test). L. The statistical results of the flow cytometry assay for CD3^+^CD8^+^ T cells (n = 6, two-tailed Student’s t test). All data were presented as mean ± SD. *, P < 0.05; **, P < 0.01; ***, P < 0.001; ****, P < 0.0001; ns, not significant.

**Figure S14. Immunotherapy response prediction of CTSB.**

Box plot shows the CTSB expression level in responders (R) and non-responders (NR) in GBM, SKCM, and NSCLC immunotherapy cohorts.
